# Long-term use of carvedilol in patients with ST-segment elevation myocardial infarction treated with primary percutaneous coronary intervention

**DOI:** 10.1371/journal.pone.0199347

**Published:** 2018-08-28

**Authors:** Hiroki Watanabe, Neiko Ozasa, Takeshi Morimoto, Hiroki Shiomi, Bao Bingyuan, Satoru Suwa, Yoshihisa Nakagawa, Chisato Izumi, Kazushige Kadota, Shigeru Ikeguchi, Kiyoshi Hibi, Yutaka Furukawa, Shuichiro Kaji, Takahiko Suzuki, Masaharu Akao, Tsukasa Inada, Yasuhiko Hayashi, Mamoru Nanasato, Masaaki Okutsu, Ryosuke Kametani, Takahito Sone, Yoichi Sugimura, Kazuya Kawai, Mitsunori Abe, Hironori Kaneko, Sunao Nakamura, Takeshi Kimura, on behalf of the CAPITAL-RCT investigators

**Affiliations:** 1 Department of Cardiovascular Medicine, Graduate School of Medicine, Kyoto University, Kyoto, Japan; 2 Department of Clinical Epidemiology, Hyogo College of Medicine, Hyogo, Japan; 3 Division of Cardiology, Juntendo University Shizuoka Hospital, Izunokuni, Japan; 4 Division of Cardiology, Tenri Hospital, Nara, Japan; 5 Division of Cardiology, Kurashiki Central Hospital, Kurashiki, Japan; 6 Division of cardiology, Shiga General Hospital, Moriyama, Japan; 7 Division of Cardiology, Yokohama City University Medical Center, Yokohama, Japan; 8 Division of Cardiology, Kobe City Medical Center General Hospital, Kobe, Japan; 9 Division of Cardiology, Toyohashi Heart Center, Toyohashi, Japan; 10 Department of Cardiology, National Hospital Organization Kyoto Medical Center, Kyoto, Japan; 11 Cardiovascular Center, Osaka Red Cross Hospital, Osaka, Japan; 12 Division of Cardiology, Tsuchiya General Hospital, Hiroshima, Japan; 13 Cardiovascular Center, Nagoya Daini Red Cross Hospital, Nagoya, Japan; 14 Division of Cardiology, Nozaki Tokushukai Hospital, Osaka, Japan; 15 Division of Cardiology, Nagoya Tokushukai General Hospital, Kasugai, Japan; 16 Division of Cardiology, Ogaki Municipal Hospital, Ogaki, Japan; 17 Division of Cardiology, Kawakita General Hospital, Tokyo, Japan; 18 Division of Cardiology, Chikamori Hospital, Kochi, Japan; 19 Division of Cardiology, Yotsuba Circulation Clinic, Matsuyama, Japan; 20 Division of Cardiology, Hoshi General Hospital, Koriyama, Japan; 21 Division of Cardiology, New Tokyo Hospital, Chiba, Japan; University of Messina, ITALY

## Abstract

**Background:**

Despite its recommendation by the current guidelines, the role of long-term oral beta-blocker therapy has never been evaluated by randomized trials in uncomplicated ST-segment elevation myocardial infarction (STEMI) patients without heart failure, left ventricular dysfunction or ventricular arrhythmia who underwent primary percutaneous coronary intervention (PCI).

**Methods and results:**

In a multi-center, open-label, randomized controlled trial, STEMI patients with successful primary PCI within 24 hours from the onset and with left ventricular ejection fraction (LVEF) ≥40% were randomly assigned in a 1-to-1 fashion either to the carvedilol group or to the no beta-blocker group within 7 days after primary PCI. The primary endpoint is a composite of all-cause death, myocardial infarction, hospitalization for heart failure, and hospitalization for acute coronary syndrome. Between August 2010 and May 2014, 801 patients were randomly assigned to the carvedilol group (N = 399) or the no beta-blocker group (N = 402) at 67 centers in Japan. The carvedilol dose was up-titrated from 3.4±2.1 mg at baseline to 6.3±4.3 mg at 1-year. During median follow-up of 3.9 years with 96.4% follow-up, the cumulative 3-year incidences of both the primary endpoint and any coronary revascularization were not significantly different between the carvedilol and no beta-blocker groups (6.8% and 7.9%, P = 0.20, and 20.3% and 17.7%, P = 0.65, respectively). There also was no significant difference in LVEF at 1-year between the 2 groups (60.9±8.4% and 59.6±8.8%, P = 0.06)

**Conclusion:**

Long-term carvedilol therapy added on the contemporary evidence-based medications did not seem beneficial in selected STEMI patients treated with primary PCI.

**Trial registration:**

CAPITAL-RCT (Carvedilol Post-Intervention Long-Term Administration in Large-scale Randomized Controlled Trial) ClinicalTrials.gov.number, NCT 01155635.

## Introduction

The long-term oral beta-blocker therapy has been recommended as class I in patients with heart failure (HF), left ventricular (LV) dysfunction, or ventricular arrhythmia after ST-segment elevation myocardial infarction (STEMI) in both the American Heart Association and the American College of Cardiology (AHA/ACC), and the European Society of Cardiology (ESC) guidelines, although randomized controlled trials supporting this recommendation were conducted before the widespread use of primary PCI for STEMI[1,2]. For uncomplicated STEMI patients without HF, LV dysfunction or ventricular arrhythmia, long-term oral beta-blocker therapy is also recommended as class I in the AHA/ACC guideline and class Ⅱa in the ESC guideline[3,4]. However, this recommendation is basically an extrapolation from the trial results in STEMI patients with HF, LV dysfunction or ventricular arrhythmia. There is no prospective trial investigating the role of long-term oral beta-blocker therapy in uncomplicated STEMI patients who underwent primary PCI. In the contemporary STEMI patients, the role of long-term oral beta-blocker therapy has been investigated only in observational studies which reported conflicting results[5–11]. Therefore, we conducted a prospective randomized trial evaluating the efficacy of long-term oral beta-blocker therapy in preventing cardiovascular events in STEMI patients with preserved LV function who underwent primary PCI.

## Materials and methods

### Study design

Carvedilol Post-Intervention Long-Term Administration in Large-scale Randomized Controlled Trial (CAPITAL-RCT) is a prospective multi-center, open-label, randomized controlled trial in 67 centers in Japan (see Acknowledgments) to investigate whether the long-term use of a beta-blocker (carvedilol) could improve long-term outcomes in STEMI patients undergoing primary PCI. Patients were eligible for the trial if they underwent successful primary PCI within 24 hours after the onset of STEMI and had preserved left ventricular ejection fraction (LVEF ≥40%) as assessed by echocardiography. Patients were ineligible for the trial if they had reduced LVEF (LVEF<40%), prior cardioverter defibrillator implantation, or contraindications to beta-blocker therapy such as unstable hemodynamic status, bradyarrhythmias, symptomatic HF, and severe bronchial asthma and/or chronic obstructive lung diseases.

After screening for eligibility and obtaining written informed consent, the enrolled patients were randomly assigned in a 1-to-1 ratio to the carvedilol group or to the no beta-blocker group within 7 days after primary PCI. Randomization was performed centrally through the electronic data capture system with a stochastic minimization algorithm to balance treatment assignment. Carvedilol was selected as the specific beta-blocker in the present study, because this agent was tested in the most recent trial evaluating the role of long-term oral beta-blocker therapy in STEMI patients (CAPRICORN trial)[2], and was the most widely used beta-blocker for STEMI patients in Japan[9]. In the carvedilol group, carvedilol was to be started from low doses and up-titrated to the target dose of 20mg daily, though the initial dose and titration of carvedilol was at the discretion of the attending physicians. The administration of other standard medications for STEMI patients such as aspirin, thienopyridines, statins, and inhibitors of the renin angiotensin system were also left to their decision.

Baseline and follow-up data were collected by the clinical research coordinators belonging to the participating centers, or the academic research organization (Research Institute for Production Development, Kyoto, Japan) (see Acknowledgments). Follow-up information on vital status, clinical events, and prescription of beta-blockers was collected by either a hospital visit or telephone contact with the patient or the referring physician at 3-month, 1-year, and final follow-up. Persistent discontinuation of carvedilol was defined as withdrawal lasting at least 2 months. Echocardiographic follow-up was recommended at 3-month and 1-year.

The study protocol (S1 Text) was approved by the institutional review board at each participating center (Juntendo University Shizuoka Hospital, Tenri Hospital, Kurashiki Central Hospital, Shiga Medical Center for Adults, Kyoto University Hospital, Yokohama City University Medical Center, Kobe City Medical Center General Hospital, Toyohashi Heart Center, National Hospital Organization Kyoto Medical Center, Osaka Red Cross Hospital, Tsuchiya General Hospital, Nagoya Daini Red Cross Hospital, Nozaki Tokushukai Hospital, Nagoya Tokushukai General Hospital, Ogaki Municipal Hospital, Kawakita General Hospital, Chikamori Hospital, Yotsuba Circulation Clinic, Hoshi General Hospital, New Tokyo Hospital, Shimada Municipal Hospital, Hiroshima City Hiroshima Citizens Hospital, Showa University Hospital, Gunma Cardiovascular Center, Kindai University Nara Hospital, Yamaguchi University Hospital, Toyama Prefectural Central Hospital, Hitachi General Hospital, National Cerebral and Cardiovascular Center, Saitama Cardiovascular and Respiratory Center, Yamagata Prefectural Central Hospital, Shizuoka City Shizuoka Hospital, Tokai University Hospital, Nagoya City East Medical Center, Kokura Memorial Hospital, Higashisumiyoshi Morimoto Hospital, Nara Prefectural Seiwa Medical Center, Tsukazaki Hospital, Kansai Electric Power Hospital, Mitsui Memorial Hospital, Fujioka General Hospital, Nihon University Itabashi Hospital, Iida Municipal Hospital, Otsu Red Cross Hospital, Fukuoka Wajiro Hospital, Fukuoka Tokushukai Hospital, Tachikawa Medical Center Tachikawa General Hospital, Miyazaki Medical Association Hospital, Saiseikai Kumamoto Hospital, Rakuwakai Otowa Hospital, Sakakibara Memorial Hospital, Wakayama Medical University Hospital, Hirosaki University Hospital, Maizuru Kyosai Hospital, Shiga University Of Medical Science Hospital, Saitama Medical Center Jichi Medical University, Japanese Red Cross Wakayama Medical Center, Toho University Ohashi Hospital, Tsukuba Medical Center Hospital, Kyoto Chubu Medical Center, Saga-ken Medical Center Koseikan, Hyogo Prefectural Amagasaki General Medical Center, Kindai University Hospital, Seirei Mikatahara General Hospital, Teikyo University Hospital, Kanazawa Cardiovascular Hospital, Kobe University Hospital). Written informed consent was obtained from all the study patients. The trial was registered with ClinicalTrials.gov.number, NCT 01155635.

### Primary and secondary endpoints

The primary endpoint was a composite of all-cause death, myocardial infarction (MI), hospitalization for acute coronary syndrome (ACS), and hospitalization for HF.

The secondary endpoints included the individual components of the primary endpoint as well as cardiac death, non-cardiac death, stroke, vasospastic angina, major bleeding, definite stent thrombosis (ST), target-lesion revascularization (TLR), and any coronary revascularization. Also, we evaluated 3 composite endpoints including cardiac death/MI/ACS/HF, cardiovascular death/MI/stroke, and death/MI/stroke/ACS/HF/any coronary revascularization. Death was regarded as cardiac in origin unless obvious non-cardiac causes could be found. MI and ST were defined according to the Academic Research Consortium definitions[12]. Stroke was defined as ischemic or hemorrhagic stroke requiring hospitalization with symptoms lasting >24 hours. ACS was diagnosed by clinical symptoms, electrocardiographic changes indicative of myocardial ischemia, and elevation of cardiac biomarkers. Unstable angina was adjudicated only in the presence of the angiographically evident culprit lesion. Hospitalizaton for HF was defined as hospitalization due to worsening heart failure requiring intravenous drug therapy. Major bleeding was defined as moderate or severe bleeding according to the Global Utilization of Stereptokinase and Tissue Plasminogen Activator for Occluded Coronary Arteries (GUSTO) classification[13]. Any coronary revascularization was defined as either PCI or CABG for any reason. Scheduled staged PCI procedures performed within 3 months of the index primary PCI were not regarded as the follow-up events, but were included in the index procedure. An independent clinical event committee adjudicated both the primary and secondary endpoints in a fashion blinded to the assigned treatment group (S1 and S2 Texts).

### Statistical analysis

The statistical analysis and reporting of the current study were conducted in accordance with the CONSORT guidelines (S2 Text). Categorical variables were expressed as number and percentage, which were compared with the chi-square test. Continuous variables were expressed as mean ± standard deviation (SD) or median with inter-quartile range (IQR) based on their distributions. The appearance of distribution was judged by illustrating the listed variables in histogram. If it was a bell-shaped appearance, the variables were judged to follow normal distribution. Continuous variables were compared using the Student’s t-test or Wilcoxon rank sum test based on their distributions, accordingly. Clinical outcomes were analyzed according to the intention-to-treat principle (ITT). The cumulative incidences of clinical events were estimated by the Kaplan-Meier method and compared by the log-rank test. Effect of the carvedilol group relative to the no beta-blocker group was assessed by the Cox proportional hazard model, and was expressed as hazard ratio (HR) with 95% confidence interval (CI). Proportional hazard assumption for the assigned group was assessed on the plots of log (time) versus log [-log (survival)] stratified by the assigned group, and the assumptions were verified to be acceptable. We only evaluated the first event for each outcome measure of interest. As a sensitivity analysis, we compared the baseline characteristics and clinical outcomes between the 2 groups of patients who received high-dose (> = 10mg) versus low-dose (<10mg) carvedilol.

The initial study protocol had sought to investigate the effect of carvedilol on mortality, because all-cause death was the primary endpoint in most of the previous trials evaluating the role of long-term oral beta-blocker therapy in STEMI patients[14–16]. With the assumption of 8.7% all-cause mortality at 3-year in STEMI patients undergoing primary PCI in a previous Japanese study, a total of 7460 patients would yield 90% power to detect superiority with a relative 23% reduction of all-cause mortality in the carvedilol group at a level of one-sided typeⅠerror of 0.025[8]. A total of 7600 patients were to be enrolled considering possible dropout during follow-up. However, due to the slow enrollment, the study protocol was modified in February 2013, changing the primary endpoint to a composite of all-cause death, MI, hospitalization for ACS, and hospitalization for HF, and reducing the target number of patients to 1300. Sample size re-calculation was based on an estimated primary event rate of 30.1% at 6-year in STEMI patients with a power of 80% to detect a relative 23% reduction of the primary endpoint in the carvedilol group as compared with the no beta-blocker group. However, the study was prematurely terminated with enrollment of 801 patients due to the slow enrollment.

All statistical analyses were conducted using JMP 12.0 (SAS Institute Inc, Cary, NC) and SAS 9.4 (SAS Institute, Inc, Cary, NC) software. All reported P values were two-sided and P values <0.05 were regarded as statistically significant.

## Results

### Study population

**Fig 1:**
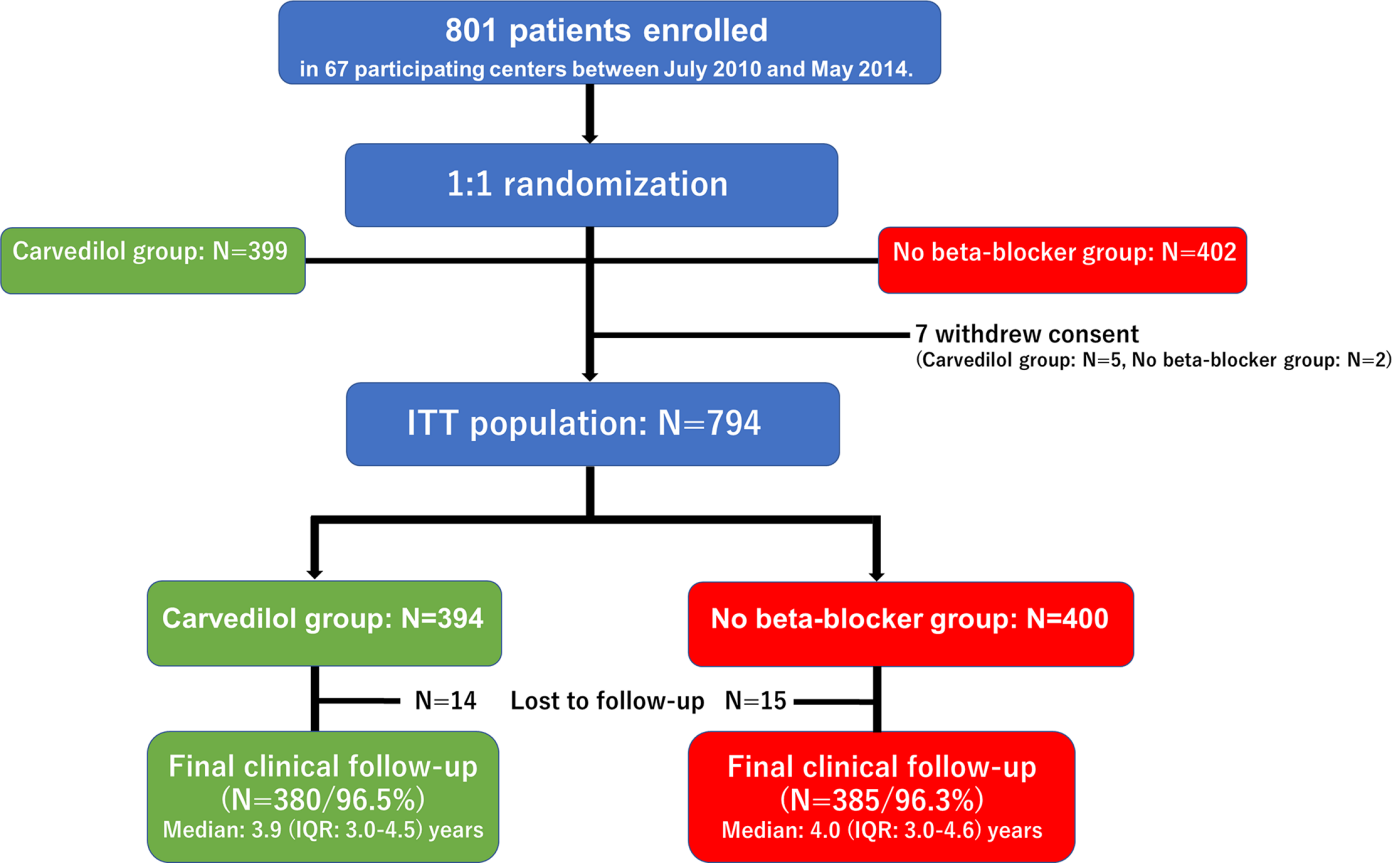
Study flow chart. ITT = intention-to-treat; IQR = interquartile range.

Between August 2010 and May 2014, a total of 801 STEMI patients were randomly assigned to the carvedilol group (N = 399) or to the no beta-blocker group (N = 402). After exclusion of 7 patients who withdrew consent, the ITT population consisted of 794 patients (carvedilol group: N = 394, and no beta-blocker group: N = 400) (Fig 1).

The baseline clinical, angiographic and procedural characteristics were well balanced between the 2 groups (Table 1). The study population represented low-risk STEMI patients as reflected by relatively young age, low Killip score, short door-to-balloon time, preserved left ventricular systolic function. Anterior STEMI accounted for nearly 40% of patients. Stents were used in almost 95% of patients with nearly identical prevalence of bare-metal and drug-eluting stents. Complete revascularization was achieved in approximately 80% of patients. The contemporary evidence-based medications such as dual antiplatelet therapy, statins, and inhibitors of the renin angiotensin system were prescribed in the majority of patients (Table 1).

**Table 1:** Baseline characteristics and medications at discharge.

Variables		Carvedilol group	No beta-blocker group
		N = 394	N = 400
**Clinical characteristics**			
	Age	63.9±11.2	64.5±11.3
	>75years	59(15%)	64(16%)
	Male	327(83%)	312(78%)
	Body mass index	24.0±3.3(N = 388)	23.9±3.2(N = 392)
	<25.0kg/m2	250/387(64%)	250/392(64%)
	Hypertension	239(61%)	234(59%)
	Dyslipidemia	191(48%)	211(53%)
	Diabetes mellitus	98(25%)	81(20%)
	Treated with insulin therapy	6(1.5%)	12(3.0%)
	Current smoking	186(47%)	188(47%)
	Prior myocardial infarction	16(4.1%)	9(2.3%)
	Prior stroke	18(4.6%)	19(4.8%)
	Peripheral artery disease	11(2.8%)	9(2.3%)
	Prior PCI	20(5.1%)	21(5.3%)
	Family history of coronary artery disease	68(17%)	81(20%)
	eGFR (ml/min/1.73m^2^)	72.4±21.5	72.8±20.9
	Hemodialysis	2(0.5%)	1(0.3%)
	Left ventricular ejection fraction	58.1±8.6(N = 390)	58.0±8.9(N = 398)
	Hemoglobin (g/dl)	13.2±1.9	13.0±2.0
	COPD	5(1.3%)	7(1.8%)
	Malignancy	29(7.4%)	23(5.8%)
**Presentation and charcteristics of STEMI**			
	Systolic blood pressure (mmHg)	121±21(N = 390)	121±20(N = 398)
	Diastolic blood pressure (mmHg)	70.2±14.8(N = 390)	71.1±14.5(N = 398)
	Heart rate (bpm)	74.8±14.3(N = 382)	73.2±13.9(N = 394)
	Location of STEMI		
	Anterior	157(40%)	153(38%)
	Inferior/Posterior	203(52%)	209(52%)
	Lateral	34(8.6%)	38(9.5%)
	Killip class		
	Ⅰ/Ⅱ	388(98%)	393(98%)
	Abnormal Q wave at admission	126(32%)	111(27%)
	Door to balloon time (minutes)	60(54–90)	66(54–102) (N = 399)
	Total ischemic time (hours)	3.8(2.5–6.2) (N = 392)	3.5(2.5–6.1) (N = 397)
	Peak CK	1893(921–3427) (N = 393)	2088(1057–3282)
**Angiographic and procedural characteristics**			
	Infarct-related artery		
	LAD	164(42%)	172(43%)
	RCA	185(47%)	185(46%)
	LCX	45(11%)	43(11%)
	Extent of coronary artery disease		
	Single-vessel disease	259(66%)	264(66%)
	Two-vessel disease	102(26%)	90(23%)
	Three-vessel disease	33(8.4%)	46(12%)
	LMCA lesion	9(2.3%)	12(3.0%)
	**Primary PCI**		
	Stent use	376(95%)	382(96%)
	BMS use	203/376(54%)	212/382(56%)
	DES use	176/376(46%)	174/382(46%)
	EES use	110/376(29%)	108/382(28%)
	BES use	22/376(5.9%)	14/382(3.7%)
	SES use	4/376(1.1%)	6/382(1.6%)
	ZES use	14/376(3.7%)	14/382(3.7%)
	Maximal stent diameter (mm)	3.2±0.5	3.2±0.5
		3(3–3.5)	3(3–3.5)
	Total stent length (mm)	24.5±13.8	25.4±13.0
		23(18–28)	23(18–28)
	Thrombus aspiration	330(84%)	322(81%)
	Distal protection	74(19%)	69(17%)
	Temporary pacemaker	33(8.4%)	37(9.3%)
	IABP use	20(5.1%)	24(6.0%)
	**Staged PCI**		
	Staged PCI for non-infarct-related artery	73/393(20%)	89/399(22%)
	Target lesion		
	LAD	40(10%)	50(13%)
	RCA	16(4.1%)	21(5.3%)
	LCX	37(9.4%)	39(9.8%)
	LMCA	2(0.5%)	4(1.0%)
	Complete revascularization	326/393(83%)	326/399(82%)
**Medication at discharge**			
	Aspirin	388(98%)	393(98%)
	Thienopyridine	379(96%)	377(94%)
	Clopidogrel	370(94%)	367(92%)
	Cilostazole	5(1.3%)	6(1.5%)
	Statin	340(86%)	345(86%)
	ACE-I/ARB	296(75%)	316(79%)
	ACE-I	163(41%)	186(47%)
	ARB	133(34%)	131(33%)
	Calcium channel blocker	54(14%)	49(12%)
	Aldosterone antagonist	38(9.6%)	35(8.8%)
	Nitrate	44(11%)	43(11%)
	Nicorandil	63(16%)	70(18%)
	Warfarin	13(3.3%)	9(2.3%)
	PPI	303(77%)	314(79%)
	H2 blocker	51(13%)	50(13%)

Continuous variables are expressed as mean ± standard deviation or median with interquartile range, and categorical variables as number (percentage). Number of patients evaluated was indicated for the variables with missing information.

PCI = percutaneous coronary intervention; eGFR = estimated glomerular filtration rate; COPD = chronic obstructive pulmonary disease; STEMI = ST-segment elevation myocardial infarction; CK = creatine phosphokinase; LAD = left anterior descending coronary artery; RCA = right coronary artery; LCX = left circumflex coronary artery; LMCA = left main coronary artery; BMS = bare-metal stents; DES = drug-eluting stents; EES = everolimus-eluting stent; BES = biolimus-eluting stent; SES = sirolimus-eluting stent; ZES = zotarolimus-eluting stent; IABP = intra-aortic balloon pumping; ACE-I = angiotensin converting enzyme inhibitors; ARB = angiotensin-receptor blockers; H2 blockers = histamine type-2 receptor blockers.

### Administration of beta-blockers

**Fig 2:**
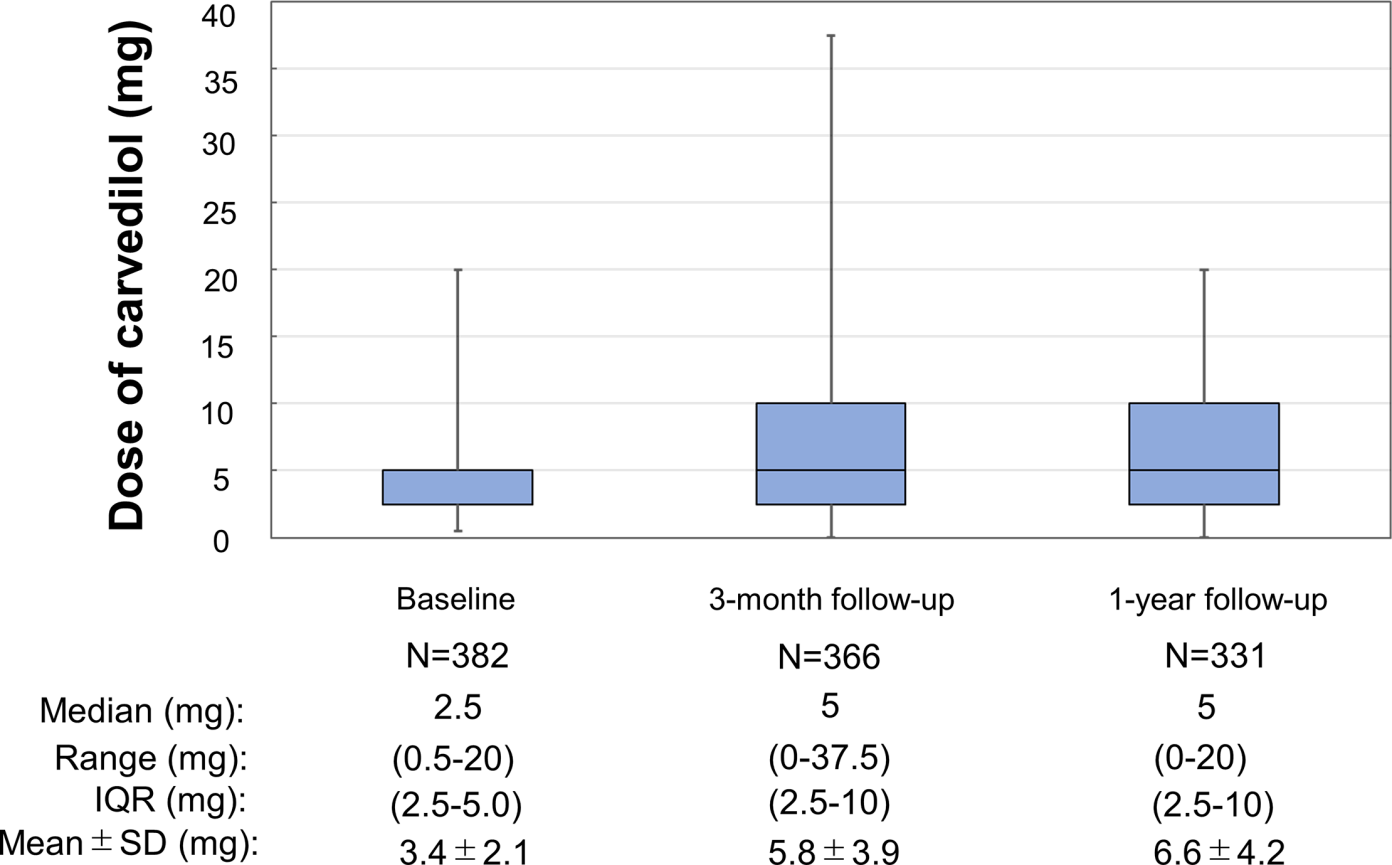
Dose of carvedilol at each follow-up. Among 394 patients in the carvedilol group, 12 patients did not receive the assigned carvedilol treatment; prescription of bisoprolol in 7 patients and no prescription of beta-blocker in 5 patients (protocol violation).

The dose of carvedilol was 3.4 ± 2.1 mg at baseline, which was up-titrated to 5.7 ± 3.9 mg at 3-month, and to 6.6 ± 4.2 mg at 1-year (Fig 2). The number of patients who took the dose of 20 mg (the maximum approved dose) was only 15 (3.8%). Prescription of carvedilol was maintained in 81% at final follow-up in the carvedilol group, while a beta-blocker was started in 14% of patients in the no beta-blocker group (S1 Fig).

### Echocardiographic follow-up

**Fig 3:**
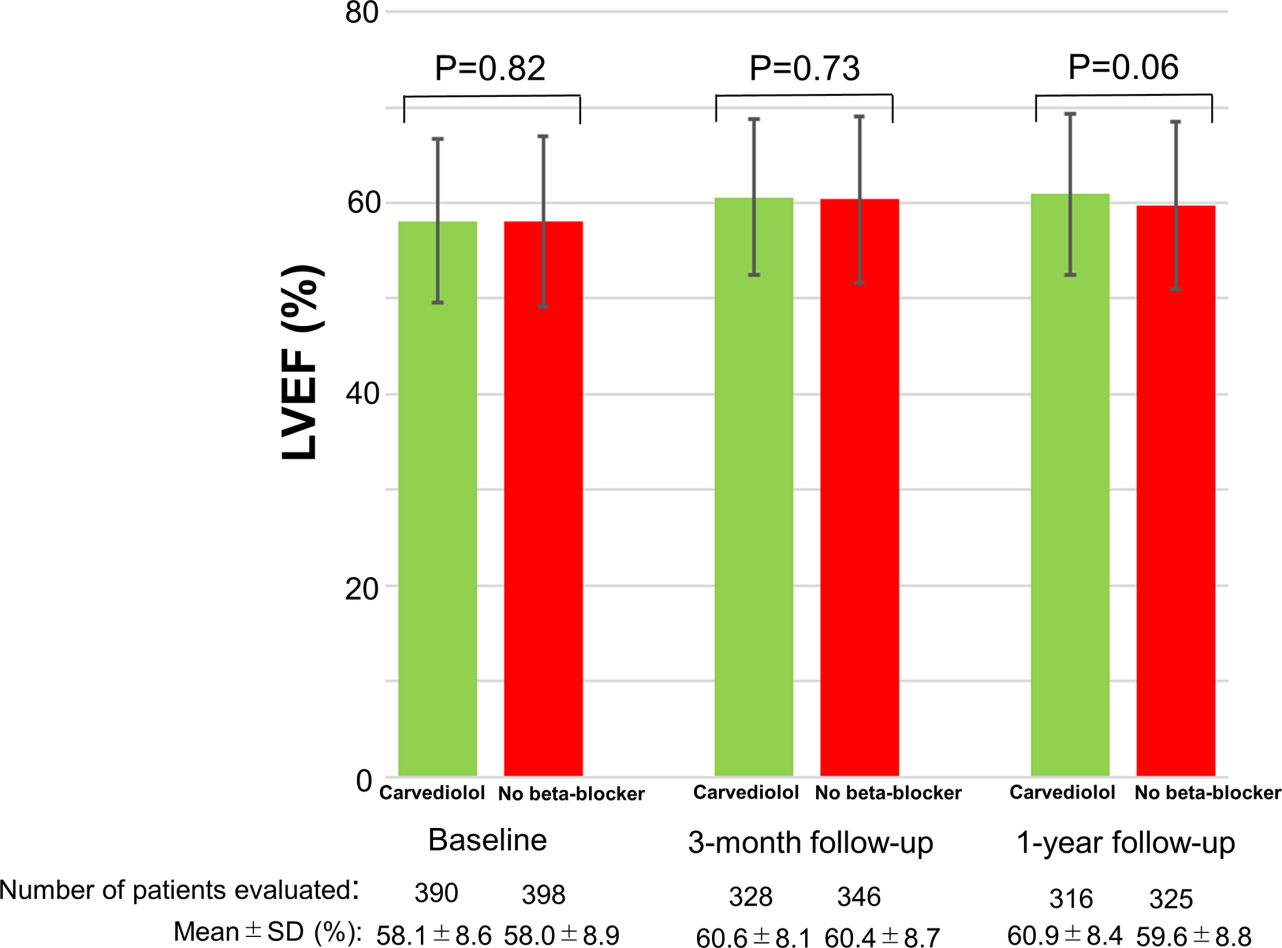
Comparison of LVEF between the carvedilol group and the no beta-blocker group at baseline, at 3-month follow-up, and at 1-year follow-up. LVEF data was missing in 6 patients (4 patients in the carvedilol group and 2 patients in the no beta-blocker group). IQR = interquartile range; SD = standard deviation, LVEF = left ventricular ejection fraction.

LVEF at baseline was well preserved in both the carvedilol and no beta-blocker groups (58.1 ± 8.6% and 58.3 ± 8.5%, P = 0.84). There was no evidence of adverse LV remodeling with well maintained LVEF at 3-month and at 1-year without any between group differences (Fig 3).

### Clinical outcomes

Final clinical follow-up beyond June 2015 was completed in 765 patients (96.4%) (carvedilol group: 380 patients [96.5%], and no beta-blocker group: 385 patients [96.3%], P = 0.88). Median follow-up period was 3.9 (IQR: 3.0–4.6) years in the overall study population (carvedilol group: 3.9 [IQR: 3.0–4.5] years, and no beta-blocker group: 4.0 [IQR: 3.0–4.6] years, P = 0.68).

**Fig 4:**
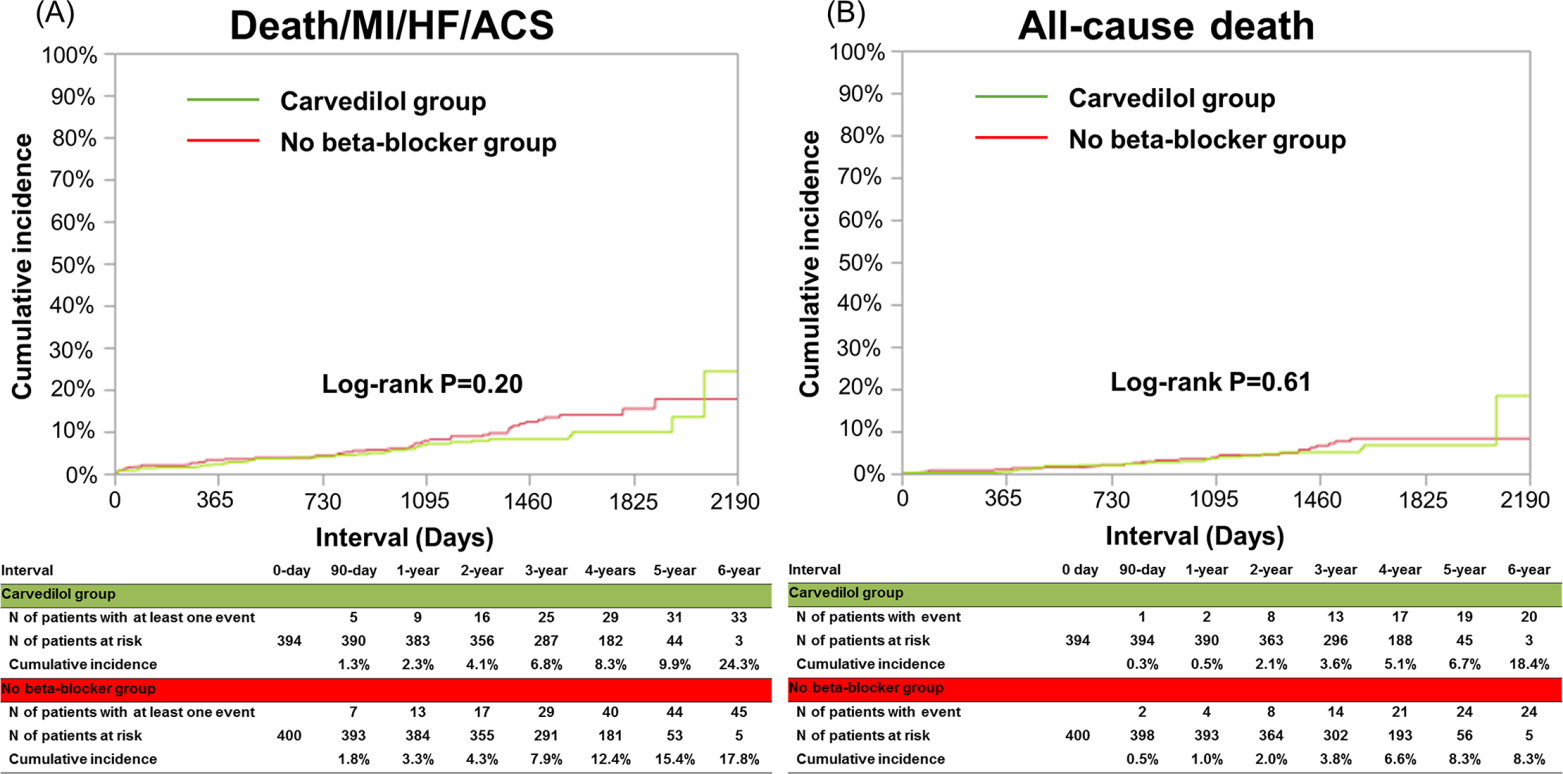
**Kaplan-Meier curves for the primary endpoint (a composite of death, MI, hospitalization for ACS, or hospitalization for HF) (A), for all-cause death (B)**.

**Fig 5:**
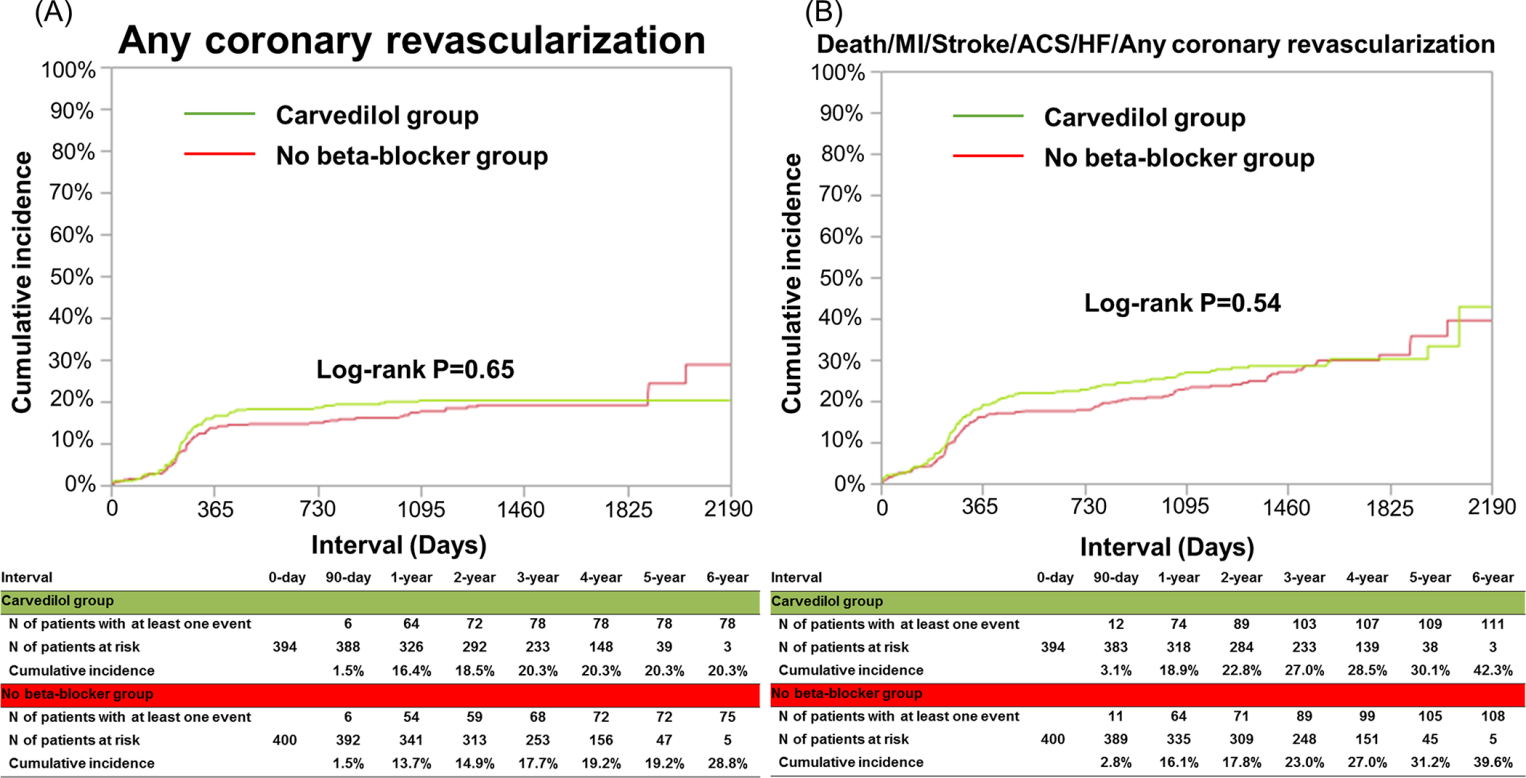
Kaplan-Meier curves for any coronary revascularization (A), and for a secondary composite endpoint (a composite of death, MI, stroke, hospitalization for ACS, hospitalization for HF or any coronary revascularization) (B). MI = myocardial infarction; ACS = acute coronary syndrome; HF = heart failure.

The cumulative 3-year incidence of the primary endpoint was not significantly different between the carvedilol and no beta-blocker groups (6.8% and 7.9%, log-rank P = 0.20, and HR: 0.75, 95%CI: 0.47–1.16, P = 0.20) (Table 2 and Fig 4). The cumulative 3-year incidences of all-cause death and cardiac death were very low and not significantly different between the 2 groups (3.6% and 3.8%, log-rank P = 0.61, and 1.1% and 1.4%, log-rank P = 0.74, respectively) (Table 2 and Fig 4). The cumulative 3-year incidences of any coronary revascularization and a composite of the primary end point or any coronary revascularization were relatively high, but not significantly different between the 2 groups (20.3% and 17.7%, log-rank P = 0.65, and 27.0% and 23.0%, log-rank P = 0.54, respectively) (Table 2 and Fig 5). There were also no significant between-group differences in other secondary endpoints (Table 2).

**Table 2:** Clinical outcomes.

		Carvedilol group	No beta-blocker group	HR(95%CI)	P value
		N of patients with events	N of patients with events		
		(Cumulative 3-year incidence)	(Cumulative 3-year incidence)		
		N = 394	N = 400		
Primary endpoint					
	Death/MI/HF/ACS	33(6.8%)	45(7.9%)	0.75(0.47–1.16)	0.20
Secondary endpoint					
	All-cause death	20(3.6%)	24(3.8%)	0.86(0.47–1.55)	0.61
	Cardiac death	6(1.1%)	5(1.4%)	1.22(0.37–4.23)	0.74
	Non-cardiac death	14(2.5%)	19(2.4%)	0.76(0.37–1.51)	0.44
	Myocardial infarction	7(2.0%)	10(1.9%)	0.71(0.26–1.84)	0.48
	Hospitalization for HF	5(1.1%)	10(1.7%)	0.51(0.16–1.46)	0.21
	Hospitalization for ACS	8(2.5%)	14(2.2%)	0.58(0.23–1.35)	0.21
	Stroke	17(4.0%)	11(2.1%)	1.59(0.75–3.50)	0.22
	Vasospastic angina	2(0.6%)	1(0.3%)	2.03(0.19–43.6)	0.55
	Major bleeding	14(2.9%)	7(1.6%)	2.07(0.86–5.46)	0.11
	Definite stent thrombosis	3(0.8%)	4(0.8%)	0.76(0.15–3.45)	0.72
	Target lesion revascularization	44(11.3%)	37(9.1%)	1.22(0.79–1.90)	0.36
	Any coronary revascularization	78(20.3%)	75(17.7%)	1.08(0.78–1.48)	0.65
	Cardiac death/MI/HF/ACS	19(4.3%)	28(5.8%)	0.69(0.38–1.22)	0.20
	Cardiovascular death/MI/stroke	27(6.8%)	26(5.3%)	1.06(0.62–1.83)	0.82
	Death/MI/HF/Stroke/ACS/HF/Any coronary revascularization	108(27.0%)	111(23.0%)	1.09(0.83–1.42)	0.54

Number of patients with event was counted through the entire follow-up period, while the cumulative incidence was indicated at 3-year.

HR = hazard ratio; CI = confidence interval; MI = myocardial infarction; ACS = acute coronary syndrome; HF = heart failure.

In a sensitivity analysis comparing the 2 groups of patients who received high-dose (> = 10mg: N = 114) versus low-dose (<10mg: N = 214) carvedilol, patients in the low-dose group were older and more often received intra-aortic balloon pumping support than those in the high-dose group (S1 Table). The cumulative 3-year incidences of the primary end point and a composite of the primary endpoint or any coronary revascularization were not significantly different between the high- and low-dose carvedilol groups (2.0% versus 4.0%, log rank P = 0.32, and 22.7% versus 24.7%, log rank P = 0.56, respectively) (S2 Table and S2 Fig).

## Discussion

The main finding of this study was that long-term carvedilol therapy added on the contemporary evidence-based medications did not reduce the primary endpoint events (a composite of all-cause death, MI, hospitalization for ACS, and hospitalization for HF) as well as a composite of the primary endpoint or any coronary revascularization in STEMI patients treated with primary PCI.

There is no previous prospective trial investigating the role of long-term oral beta-blocker therapy in STEMI patients who underwent primary PCI. In the contemporary STEMI patients, there were several observational studies evaluating the role of long-term oral beta-blocker therapy with conflicting results[5–11]. In a metaanalysis of 10 observational studies including 40873 patients, use of beta-blockers was associated with a reduced risk of all-cause death, but the benefit of beta-blockers in preventing all-cause death was restricted to those with reduced ejection fraction, those with low use proportion of other secondary prevention drugs or those with non-ST-segment elevation myocardial infarction[17]. On the other hands, another metaanalysis of 7 observational studies including 10857 patients suggested that oral beta-blocker therapy is associated with decreased all-cause mortality in patients with STEMI who are treated with primary PCI and who have preserved LVEF[18]. However, there is a possibility of profound selection bias in observational studies by the trend toward avoiding the use of beta-blockers in sicker patients who are deemed to have a poor prognosis. Therefore, the true efficacy of beta-blockers in reducing cardiovascular events could only be addressed by the randomized controlled trials. This is the first randomized trial in the primary PCI era assessing the effect of long-term beta-blocker therapy in STEMI patient undergoing primary PCI, suggesting no significant benefit of a beta-blocker in reducing the cardiovascular events. Regrettably, we could not draw a definitive conclusion from the current study, because we could not deny a moderate effect of carvedilol due to insufficient power of the study for the primary endpoint. However, there are several important implications from our study. First, mortality was the primary endpoint in most of the previous randomized trials of beta-blockers in STEMI patients. However, long-term mortality rate, particularly cardiac death rate, was very low in the present study (annual rate of 1.3% for all-cause death and 0.47% for cardiac death during 3-year after STEMI in the control group). In contrast, the annual mortality rate was 4.4%, and the majority of death was due to cardiovascular causes (91% in the placebo group) in the ß-Blocker Heart Attack Trial (B-HAT), which was conducted between 1978 and 1980 to test clinical benefits of long-term administration of propranolol for patients with acute myocardial infarction[14]. This relatively high cardiovascular mortality was consistently observed in other trials conducted before the primary PCI era[1,14–16]. Recent several studies suggested that the cause of death during long-term follow-up of STEMI patients shifted from cardiac to non-cardiac disease, which coincided with the finding that non-cardiac death accounted for almost two-thirds of all-cause death in the present study[19,20]. Given the extremely low rate of cardiac death in contemporary uncomplicated STEMI patients, it is very unlikely for the long-term beta-blocker therapy to provide a large absolute reduction in mortality. Second, one of the mechanisms for the benefits of the beta-blocker therapy might be the attenuation of myocardial oxygen demand, which might lead to reduction of coronary revascularization procedure. However, there was no significant between groups difference in the rate of any coronary revascularization, which was one of the secondary endpoint with higher incidence. Third, another mechanism for the benefits of the beta-blocker therapy might be the prevention of cardiotoxic effects of catecholamines, leading to the inhibition of the adverse LV remodeling in patients with low LVEF[21,22]. In the current study, serial echocardiographic evaluation was recommended by the protocol, and LVEF was well maintained at 3-month and at 1-year without evidence of adverse LV remodeling regardless of carvedilol administration (Fig 2B). Carvedilol is unlikely to improve the long-term prognosis of STEMI patients without favorable influence on the remodeling process.

### Limitations

This trial has several important limitations. First of all, the current trial was underpowered to detect even a moderate difference in the treatment effect for the primary endpoint. The final sample size was reduced due to the slow enrollment, because administration of beta-blockers in STEMI patients is the guideline recommended therapy, and therefore, conducting a randomized trial in this field was very challenging. Nevertheless, we did not find any significant between groups difference in a composite of the primary endpoint or coronary revascularization, which was an adequately powered and clinically relevant endpoint, suggesting that long-term oral carvedilol therapy was not associated with discernible clinical benefits. Second, there was some separation of the Kaplan-Meier curves for the primary endpoint beyond 3-year favoring the carvedilol group. However, in the previous randomized trials, the benefit of beta-blockers in reducing death and/or MI was evident within 1-year[2,14]. Therefore, it is unlikely for carvedilol to have delayed beneficial effects beyond 3-year without any effects during the initial 3 years. Actually, the AHA/ACCF secondary prevention guidelines recommend a 3-year treatment course for beta-blocker in uncomplicated STEMI patients[3]. Third, the open-label trial design is another limitation of the study. However, the initiation of beta-blockers in the no beta-blocker group was acceptably low. Fourth, the mean maintenance dose of carvedilol in the current trial was only 6.3mg/day at 1-year, which might have attenuated the potential benefits of carvedilol. However, a study by Goldberger et al. evaluated the association of the dose of beta-blocker with survival after acute myocardial infarction, demonstrating that the lowest mortality was observed at 25% of the target dose (metoprolol 50 mg/day)[23]. In the sensitivity analysis of the present study, clinical outcomes were not different between the high- and low-dose carvedilol groups. The relationship between the doses of beta-blocker and the magnitude of clinical benefits is still debated[21,24–26]. In our previous report from a Japanese real world registry of 3692 STEMI patients, oral beta-blockers were prescribed only in 44% of patients, and the daily dose of carvedilol was median 5 (interquartile range: 2–10) mg, which was comparable to the dose used in the present study(9). Taken together, the carvedilol dose lower than that used outside Japan is a big limitation to extrapolate the present study results outside Japan. Nevertheless, carvedilol at the dose used in daily Japanese clinical practice seems not to reduce cardiovascular events in uncomplicated STEMI patients treated with primary PCI. Finally, the present study patients could be a highly selected low-risk STEMI population with mean LVEF of 60%. It is unknown whether the present trial result could be applicable to those uncomplicated STEMI patients with lower LVEF. However, in the contemporary registries of STEMI patients treated with primary PCI, the majority of patients have well preserved LVEF and the rates of cardiovascular events such as cardiac death, MI and HF are remarkably low[27–29].

### Conclusions

Long-term carvedilol therapy added on the contemporary evidence-based medications did not seem beneficial in selected STEMI patients treated with primary PCI.

## Supporting information

S1 Table Baseline characteristics and medications at discharge in the high-dose and the low-dose subgroups. (DOCX)

S2 Table Clinical outcomes in the high-dose and the low-dose subgroups. (DOCX)

S1 Fig Kaplan-Meier curves for the cumulative incidences of (A) persistent discontinuation of carvedilol in the carvedilol group and of (B) initiation of beta-blockers in the no beta-blocker group.
(DOCX)

S2 Fig Kaplan-Meier curves for the cumulative incidences of (A) the composite of all-cause death, myocardial infarction, heart failure hospitalization and emergent hospitalization for acute coronary syndrome and of (B) the composite of all-cause death, myocardial infarction, stroke, heart failure hospitalization, emergent hospitalization for acute coronary syndrome and any coronary revascularization in the high-dose and the low-dose groups.
(DOCX)

S1 Text Study protocol. (DOCX)

S2 Text CONSORT checklist. (DOCX)
