# Supplementary material for: Long-term use of carvedilol in patients with ST-segment elevation myocardial infarction treated with primary percutaneous coronary intervention
Source: PLoS One. 2018 Aug 28;13(8):e0199347. doi: 10.1371/journal.pone.0199347 (PMC6112626; doi:10.1371/journal.pone.0199347)
Supplement: S1 Table — (DOCX) [file pone.0199347.s001.docx]

**S1 Table: Baseline Characteristics and Medications at Discharge in the high-dose and the low-dose subgroups**

| Variables | | High-dose group | Low-dose group | P value |
| --- | --- | --- | --- | --- |
|  |  | N=114 | N=214 |  |
| **Clinical characteristics** | |  |  |  |
|  | Age | 61.4±10.1 | 64.5±11.3 | 0.02 |
|  | >75years | 9(7.9%) | 34(16%) | 0.03 |
|  | Male | 99(87%) | 174(81%) | 0.19 |
|  | Body mass index | 24.3±3.１(N=113) | 23.9±3.４(N=211) | 0.29 |
|  | <25.0kg/m2 | 72/113(64%) | 138/211(65%) | 0.76 |
|  | Hypertension | 68(60%) | 123(57%) | 0.70 |
|  | Dyslipidemia | 55(48%) | 102(48%) | 0.92 |
|  | Diabetes mellitus | 28(25%) | 49(23%) | 0.74 |
|  | Treated with insulin therapy | 1(0.9%) | 4(1.9%) | 0.47 |
|  | Current smoking | 55(48%) | 99(46%) | 0.73 |
|  | Prior myocardial infarction | 2(1.8%) | 8(3.7%) | 0.30 |
|  | Prior stroke | 4(3.5%) | 8(3.7%) | 0.92 |
|  | Peripheral artery disease | 3(2.6%) | 7(3.3%) | 0.75 |
|  | Prior PCI | 2(1.8%) | 11(5.1%) | 0.11 |
|  | Family history of coronary artery disease | 25(22%) | 31(14%) | 0.09 |
|  | eGFR (ml/min/1.73m²) | 73.8±23.0 | 71.8±21.0 | 0.44 |
|  | Hemodialysis | 0 | 1(0.5%) | 0.35 |
|  | Left ventricular ejection fraction | 57.2±8.7 | 58.3±8.4（N=213) | 0.23 |
|  | Hemoglobin (g/dl) | 13.4±1.8 | 13.2±2.0 | 0.28 |
|  | COPD | 1(0.9%) | 3(1.4%) | 0.67 |
|  | Malignancy | 7(6.1%) | 15(7.0%) | 0.76 |
| **Presentation and characteristics of STEMI** | |  |  |  |
|  | Systolic blood pressure (mmHg) | 123±20(N=112) | 121±20 | 0.33 |
|  | Diastolic blood pressure (mmHg) | 71±14(N=112) | 71±15 | 0.82 |
|  | Heart rate (bpm) | 76±14(N=111) | 75±15(N=207) | 0.41 |
|  | Location of STEMI |  |  | 0.46 |
|  | Anterior | 40(35%) | 90(42%) |  |
|  | Inferior/Posterior | 63(55%) | 107(50%) |  |
|  | Lateral | 11(9.7%) | 17(7.9%) |  |
|  | Killip class |  |  |  |
|  | Ⅰ/Ⅱ | 113(99%) | 211(99%) | 0.67 |
|  | Abnormal Q wave at admission | 39(34%) | 71(33%) | 0.85 |
|  | Door to balloon time(minutes) | 60(48-90) | 66(54-90) | 0.04 |
|  | Total ischemic time(hours) | 3.5(2.45-6.4) | 3.7(2.4-5.9) (N=212) | 0.99 |
|  | Peak CK | 2249(999.5-3806) | 1701(860-3244) | 0.047 |
| **Angiographic and procedural characteristics** | |  |  |  |
|  | Infarct-related artery |  |  | 0.60 |
|  | LAD | 43(38%) | 93(43%) |  |
|  | RCA | 57(12%) | 98(46%) |  |
|  | LCX | 14(50%) | 23(11%) |  |
|  | Extent of coronary artery disease |  |  | 0.84 |
|  | Single-vessel disease | 77(68%) | 139(65%) |  |
|  | Two-vessel disease | 29(25%) | 61(29%) |  |
|  | Three-vessel disease | 8(7.0%) | 14(6.5%) |  |
|  | LMCA lesion | 2(1.8%) | 5(2.3%) | 0.72 |
|  | **Primary PCI** |  |  |  |
|  | Stent use | 106(93%) | 208(97%) | 0.08 |
|  | BMS use | 62/106(58%) | 107/208(51%) | 0.24 |
|  | DES use | 46/106(43%) | 101/208(49%) | 0.39 |
|  | EES use | 30/106(28%) | 63/208(30%) | 0.71 |
|  | BES use | 5/106(2.8%) | 16/208(7.7%) | 0.30 |
|  | SES use | 0 | 3/208(1.4%) | 0.11 |
|  | ZES use | 3/106(2.8%) | 9/208(4.3%) | 0.50 |
|  | Maximal stent diameter (mm) | 3.5(3-3.5) | 3(3-3.5) | 0.03 |
|  |  | 3.3±0.5 | 3.2±0.5 |  |
|  | Total stent length (mm) | 24(18-28.5) | 20(16-28) | 0.02 |
|  |  | 26.8±15.6 | 23.5±13.1 |  |
|  | Thrombus aspiration | 95(83%) | 178(83%) | 0.97 |
|  | Distal protection | 42(20%) | 21(18%) | 0.79 |
|  | Temporary pacemaker | 10(8.8%) | 16(7.5%) | 0.68 |
|  | IABP use | 2(1.8%) | 14(6.5%) | 0.04 |
|  | **Staged PCI** |  |  |  |
|  | Staged PCI for non-infarct-related artery | 20(18%) | 41/213(19%) | 0.71 |
|  | Target lesion |  |  |  |
|  | LAD | 10(8.8%) | 21(9.8%) | 0.76 |
|  | RCA | 6(5.3%) | 6(2.8%) | 0.27 |
|  | LCX | 8(7.0%) | 21(9.8%) | 0.39 |
|  | LMCA | １(0.9%) | 0 | 0.15 |
|  | Complete revascularization | 90(79%) | 171/213(80%) | 0.78 |
| **Medication at discharge** | |  |  |  |
|  | Aspirin | 113(99%) | 212(99%) | 0.96 |
|  | Thienopyridine | 111(97%) | 209(98%) | 0.87 |
|  | Clopidogrel | 111(97%) | 201(94%) | 0.15 |
|  | Cilostazole | 1(0.9%) | 3(1.4%) | 0.67 |
|  | Statin | 103(90%) | 183(86%) | 0.20 |
|  | ACE-I/ARB | 80(70%) | 166(78%) | 0.14 |
|  | ACE-I | 52(46%) | 90(42%) | 0.54 |
|  | ARB | 28(25%) | 76(36%) | 0.04 |
|  | Calcium channel blocker | 17(15%) | 31(14%) | 0.92 |
|  | Aldosterone antagonist | 6(5.3%) | 23(11%) | 0.08 |
|  | Nitrate | 13(11%) | 27(13%) | 0.75 |
|  | Nicorandil | 19(17%) | 33(15%) | 0.77 |
|  | Warfarin | 5(4.4%) | 6(2.8%) | 0.46 |
|  | PPI | 89(78%) | 169(79%) | 0.85 |
|  | H2 blocker | 13(11%) | 23(11%) | 0.86 |

Continuous variables are expressed as mean ± standard deviation or median with interquartile range, and categorical variables as number (percentage). Number of patients evaluated was indicated for the variables with missing information.

PCI=percutaneous coronary intervention; eGFR=estimated glomerular filtration rate; COPD=chronic obstructive pulmonary disease; STEMI=ST-segment elevation myocardial infarction; CK=creatine phosphokinase; LAD=left anterior descending coronary artery; RCA=right coronary artery; LCX=left circumflex coronary artery; LMCA=left main coronary artery; BMS=bare-metal stents; DES=drug-eluting stents; EES=everolimus-eluting stent; BES=biolimus-eluting stent; SES=sirolimus-eluting stent; ZES=zotarolimus-eluting stent; IABP=intra-aortic balloon pumping; ACE-I=angiotensin converting enzyme inhibitors; ARB=angiotensin-receptor blockers; H2 blockers=histamine type-2 receptor blockers.
