# Supplementary material for: Long-term use of carvedilol in patients with ST-segment elevation myocardial infarction treated with primary percutaneous coronary intervention
Source: PLoS One. 2018 Aug 28;13(8):e0199347. doi: 10.1371/journal.pone.0199347 (PMC6112626; doi:10.1371/journal.pone.0199347)
Supplement: S2 Table — (DOCX) [file pone.0199347.s002.docx]

**S2 Table: Clinical Outcomes in the high-dose and the low-dose subgroups**

| Variables | **High-dose group** | **Low-dose group** | Crude HR(95%CI) | P value |
| --- | --- | --- | --- | --- |
|  | N of patients with events | N of patients with events |  |  |
|  | (Cumulative 3-year incidence) | (Cumulative 3-year incidence) |  |  |
|  | N=114 | N=214 |  |  |
| Death/MI/HF/ACS | 2(2.0%) | 8(4.0%) | 0.53(0.12-1.70) | 0.30 |
| Death/MI/HF/Stroke/ACS/Any coronary revascularization | 25(22.7%) | 52(24.7%) | 0.87(0.54-1.37) | 0.56 |

Number of patients with event was counted through the entire follow-up period, while the cumulative incidence was indicated at 3-year.

HR=hazard ratio; CI=confidence interval; MI=myocardial infarction; ACS=acute coronary syndrome; HF=heart failure.
