# Supplementary material for: Long-term use of carvedilol in patients with ST-segment elevation myocardial infarction treated with primary percutaneous coronary intervention
Source: PLoS One. 2018 Aug 28;13(8):e0199347. doi: 10.1371/journal.pone.0199347 (PMC6112626; doi:10.1371/journal.pone.0199347)
Supplement: S1 Fig — Kaplan-Meier curves for the cumulative incidences of (A) persistent discontinuation of carvedilol in the carvedilol group and of (B) initiation of beta-blockers in the no beta-blocker group. (DOCX) [file pone.0199347.s003.docx]

**Supporting Figure titles and legends**

**S1 Figure:** Kaplan-Meier curves for the cumulative incidences of (A) persistent discontinuation of carvedilol in the carvedilol group and of (B) initiation of beta-blockers in the no beta-blocker group.

Among 394 patients in the carvedilol group, we excluded 12 patients from this analysis, in whom carvedilol was not administered after randomization.

**S1 Figure**

**(A) (B)**

**
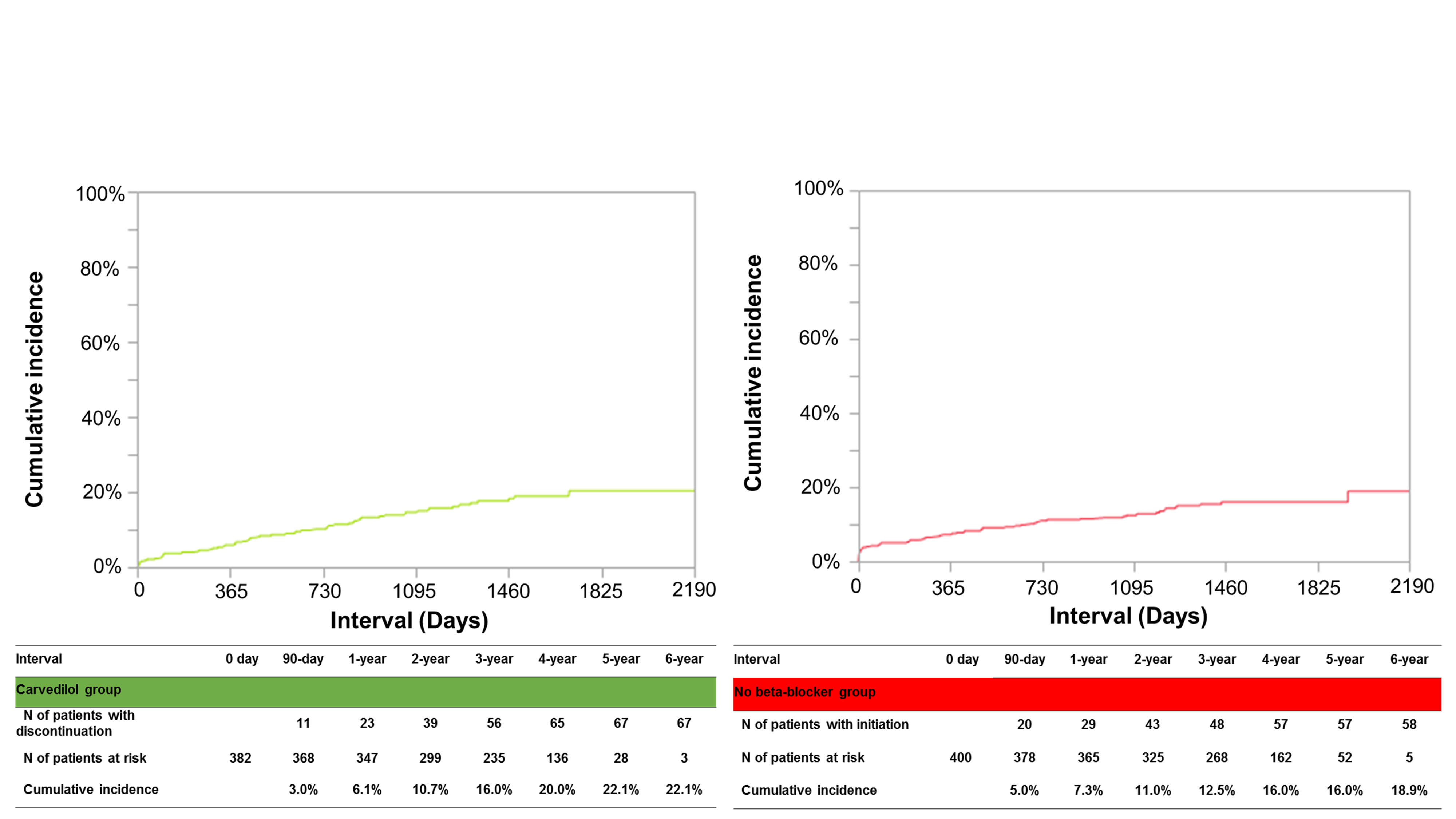
**
