# Supplementary material for: Long-term use of carvedilol in patients with ST-segment elevation myocardial infarction treated with primary percutaneous coronary intervention
Source: PLoS One. 2018 Aug 28;13(8):e0199347. doi: 10.1371/journal.pone.0199347 (PMC6112626; doi:10.1371/journal.pone.0199347)
Supplement: S2 Fig — Kaplan-Meier curves for the cumulative incidences of (A) the composite of all-cause death, myocardial infarction, heart failure hospitalization and emergent hospitalization for acute coronary syndrome and of (B) the composite of all-cause death, myocardial infarction, stroke, heart failure hospitalization, emergent hospitalization for acute coronary syndrome and any coronary revascularization in the high-dose and the low-dose groups. (DOCX) [file pone.0199347.s004.docx]

**Supporting Figure titles and legends**

**S2 Figure:** Kaplan-Meier curves for the cumulative incidences of (A) the composite of all-cause death, myocardial infarction, heart failure hospitalization and emergent hospitalization for acute coronary syndrome and of (B) the composite of all-cause death, myocardial infarction, stroke, heart failure hospitalization, emergent hospitalization for acute coronary syndrome and any coronary revascularization in the high-dose and the low-dose groups.

**S2 Figure**

**(A) (B)**

**
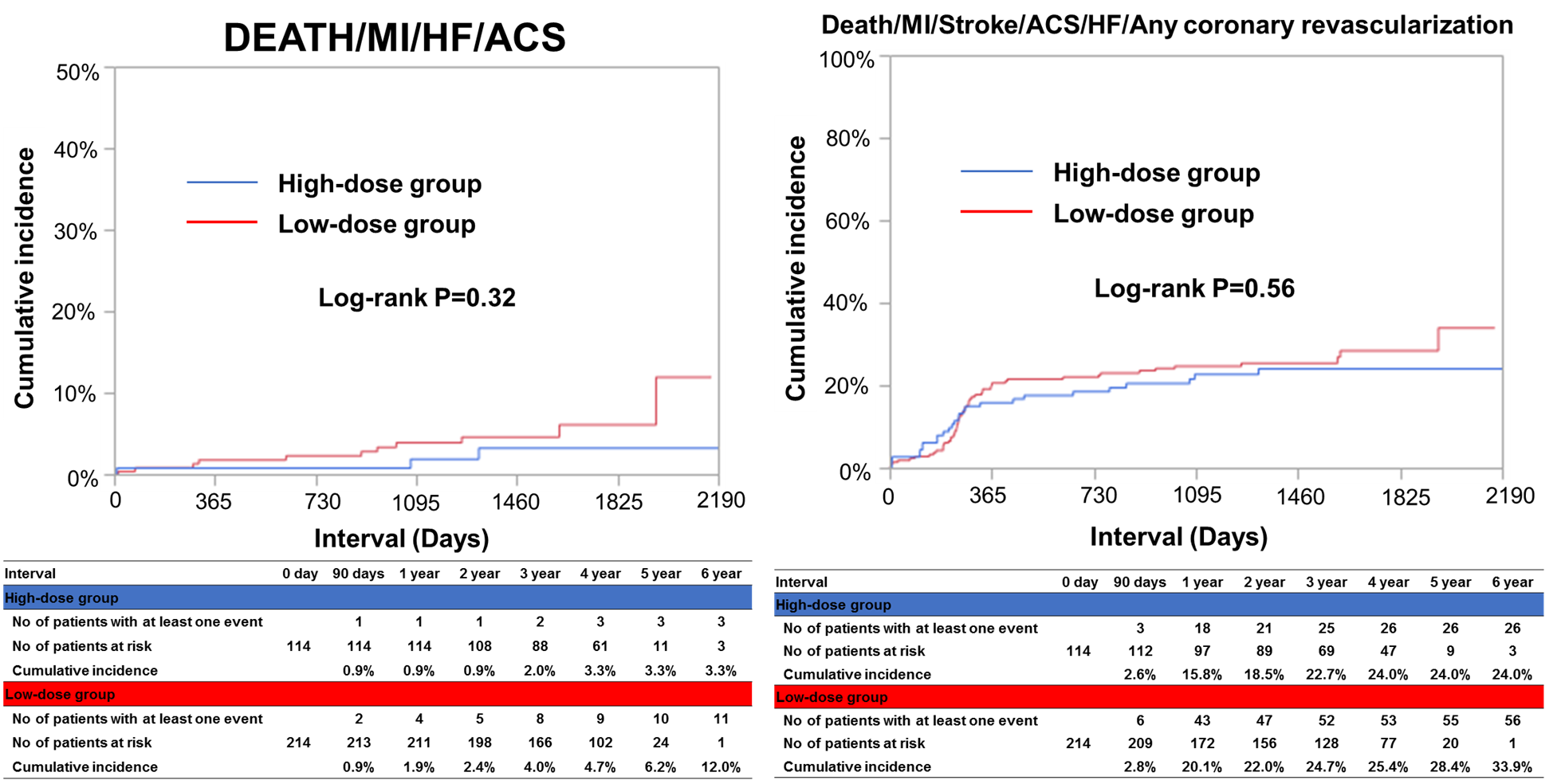
**
