# Supplementary material for: Long-term use of carvedilol in patients with ST-segment elevation myocardial infarction treated with primary percutaneous coronary intervention
Source: PLoS One. 2018 Aug 28;13(8):e0199347. doi: 10.1371/journal.pone.0199347 (PMC6112626; doi:10.1371/journal.pone.0199347)
Supplement: S1 Text — (DOCX) [file pone.0199347.s005.docx]

**Protocol**

**Final protocol and Statistical Analytic Plan: Date: 18/02/2013**

**Long-term Use of Beta-blocker in Patients with ST-segment Elevation Myocardial Infarction Treated with Primary Percutaneous Coronary Intervention: *CA*rvedilol *P*ost-*I*ntervention Long-*T*erm *A*dministration in *L*arge-scale *R*andomized *C*ontrolled *T*rial)**

**CAPITAL-RCT**

**Principal investigator:**

Takeshi Kimura, MD, PhD

Department of Cardiovascular Medicine, Graduate School of Medicine, Kyoto University

**Address for principle investigator**

Takeshi Kimura, MD, PhD

Address: 54 Shogoin-Kawahara-cho, Sakyo-ku, Kyoto, 606-8507, Japan

Department of Cardiovascular Medicine, Kyoto University

TEL: +81-75-751-4255/FAX: +81-75-751-3299

Email address: [taketaka@kuhp.kyoto-u.ac.jp](mailto:taketaka@kuhp.kyoto-u.ac.jp)

Contents

**０.** Schematic illustration

Ⅰ. Summary

II. Background and purpose of the trial

III. Trial plan

1. Study design
2. Study period and enrollment period
3. Selection of study participant
4. Enrollment
5. Intervention (study drug)
6. Follow-up
7. Schedule of follow-up
8. Premature discontinuation and dropout
9. Statistical analysis
10. Adverse events
11. Expense and honorarium
12. Ethical issues
13. Approval and revision of protocol
14. Discontinuation and termination of the study
15. Presentation and publication of the study result
16. Handling of the published study
17. Funding source
18. Study organization
19. Reference

０．Schematic illustration

Patients who underwent successful primary PCI within 24 hours after the STEMI onset

Patients with LVEF > 40% evaluated by echocardiography

Ineligible patients

Patients who do not meet eligibility criteria of the trial

Informed consent to randomized controlled trial

If candidates meet eligibility criteria of the trial, they are randomized through the Web system

Randomization

（within 7 days after the onset）

Carvedilol group (n=3800)

No beta-blocker group (n=3800)

**Ｉ．Summary of the trial plan**

Data collection and analysis

　Follow-up（3 years）

**1．Objective**

This is a physician-initiated, multi-center, open-label, randomized controlled trial (RCT), which investigates the clinical effect of β blocker on the long-term prognosis in patients with ST-elevation acute myocardial infarction (STEMI) undergoing primary percutaneous coronary intervention (PCI) during longer follow-up period.

1. **Background**

Previous large-scale studies performed in 1960’s to 1980’s demonstrated that the early administration of β blocker improved the prognosis of STEMI patients before the introduction of reperfusion therapy or treated with fibrinolysis. The guidelines also recommend β blocker as one of the first-line drugs for secondary prevention in STEMI patients. However, as there is no prospective RCT, which evaluates the clinical efficacy of β blocker in STEMI patients undergoing primary PCI, it is still unknown whether β blocker is beneficial in this subcategory of patients. In Japan, primary PCI is more frequently performed for STEMI patients than in foreign countries and the proportion of STEMI patients, in whom β blocker is prescribed in clinical practice, is remarkably low. Hence, we should reconfirm the role of β blocker as one of the standard medications in STEMI patients who underwent primary PCI.

**3．Design**

Multi-center, prospective, open-label, randomized controlled study

**4．Study population and Method**

The study enrollment goal is 1300 patients in total (Carvedilol group 650 patients/No-β blocker group 650 patients). Patients are eligible if they undergo successful primary PCI within 24 hours after the onset of STEMI and have preserved left ventricular ejection fraction >40% assessed by echocardiography after the date of 7/13/2010. Enrolled patients are randomized in a 1 to 1 fashion to the carvedilol group or to the no β blocker group within seven days after the primary PCI unless they fulfill any exclusion criteria. The β blocker used in this trial is carvedilol. The initial dose and up-titration of carvedilol is left to the physician in charge and the maximum dose is 20mg. Administration of other standard medications such as aspirin, statins, angiotensin-converting enzyme inhibitors is also left to the physician in charge.

1. **Endpoint**
   1. Primary endpoint

1) Death from any cause（All-cause death）

2) A composite of all-cause death, myocardial infarction, hospitalization for acute coronary syndrome, hospitalization for heart failure

5-2 Secondary endpoint：evaluated in the hierarchical order

1) A composite of all-cause death, myocardial infarction, hospitalization for acute coronary syndrome, and hospitalization for congestive heart failure

2) Cardiac death

3) Sudden cardiac death

4) Cardiovascular death

5) Myocardial infarction (MI)

6) Hospitalization for acute coronary syndrome

7) Persistent ventricular tachycardia and ventricular fibrillation

8) Hospitalization for congestive heart failure

9) ARC-definite stent thrombosis

10) Any target-lesion revascularization

11) Any clinically-driven target lesion revascularization

12) Any coronary revascularization

13) Any clinically-driven coronary revascularization

14) CABG

15) Stroke

16) Vasospastic angina

17) Major bleeding

18) A composite of all-cause death, MI, stroke, hospitalization for acute coronary syndrome, hospitalization for congestive heart failure, and any coronary revascularization

19) A composite of cardiac death, MI, hospitalization for acute coronary syndrome, and hospitalization for congestive heart failure

20) A composite of cardiovascular death, MI, and stroke

**II．Background and Objective**

Patients with STEMI, which is caused by the occlusion of coronary artery perfusing the myocardium, are generally in a more serious condition and treated differently from non-STEMI patients. The most important factor, which determines the short and the long-term prognosis of STEMI patients proved to be the restoration of coronary artery flow. Particularly, it was reported that primary PCI within 90 minutes after the hospital arrival was essential because the shorter interval from the onset to the initiation of primary PCI was associated with lower mortality^1^. Successful reperfusion not only affects the prognosis, but also might change the needs for other medications in STEMI patients.

Several large-scale studies in 1960’s to 1980’s, before the establishment of primary PCI, showed that the early administration of β blocker to patients with acute myocardial infarction (AMI) shrinked the size of infarcted myocardium, resulting in a reduced rate of death, re-infarction, cardiac rupture, ventricular fibrillation, supraventricular arrhythmias^2–5^. Several studies indicated use of β blocker diminished the rate of re-infarction and recurrence of ischemia even in STEMI patients treated by fibrinolytic therapy and that intravenous administration of β blocker in the acute phase (within 2 hours after the onset) reduced mortality^6–10^. The hypothesis underlying this result is that the early administration of β blocker reduces heart rate, blood pressure and myocardial contractility, which leads to decreased myocardial oxygen demand and less infarcted myocardium; it reduces complications associated with AMI, re-infarction and fatal arrhythmias. Moreover, it is possible that the reduction of heart rate prolongs the diastolic interval, which improves the perfusion of injured myocardium mainly in subendocardium. BHAT and CAPRICORN reported that β blocker therapy in the recovery phase was effective as secondary prevention of ischemic events in cases without β blocker therapy in the very acute phase^3,11^. It was confirmed by many RCTs that β blocker therapy improved the prognosis of patients with decreased LV function or with heart failure and RCT was already reported in AMI patient, too^11–13^. In contrast, there has been no RCT, which assesses mortality improvement by β blocker therapy in STEMI patients with preserved LV function without residual ischemia after primary PCI. Nevertheless, the current clinical guideline recommends the administration of β blocker within 24 to 48 hours after the onset to all the STEMI patients regardless of the extent of LV function or residual ischemia unless they are contraindicated to β blocker^14,15^. If the mortality benefit of β blocker therapy is based on the reduction of myocardial oxygen demand and the improvement of coronary perfusion in the injured myocardium, there is a possibility that β blocker therapy has little clinical effect on patients with preserved LV function or with no residual ischemia who underwent complete revascularization by primary PCI.

However, when we discuss treatment of STEMI patients, it is necessary to consider that the medical circumstances in Japan are different from those in Western countries. There are fewer institutions where primary PCI can be carried out in Western countries and patients are dispersed in local areas. Therefore, as it is sometimes impossible to transfer STEMI patients to facilities where primary PCI is possible, intravenous thrombolytic therapy is often conducted as reperfusion therapy whereas primary PCI is not so often performed. On the contrary, it is possible to deliver most STEMI patients to such facilities in Japan, which has approximately 1500 hospitals where primary PCI is available. Actually, if patients visit emergency department and are diagnosed as having AMI, more than half of them receive primary PCI here^16^. In spite of a large difference between the countries in the frequency of primary PCI for STEMI patients, the clinical guideline by Japanese Circulation Society, conforming to the European and American guidelines, recommends the early administration of β blocker to STEMI patients with no contraindication to it regardless of whether primary PCI is performed or not. However, because no RCT has ever investigated the efficacy of β blocker in STEMI patients with preserved LV ejection fraction (LVEF) or with little residual ischemia who undergo primary PCI, further research should be warranted to elucidate it. The JBCMI study, which was recently reported by Ogawa et al. in Kumamoto Univ., pointed out that the long-term use of β blocker did not improve the prognosis of Japanese STEMI patients as compared with the use of calcium-channel blocker. In the JBCMI study, 1090 AMI patients within 30 days after the onset were randomized in 1 to 1 fashion to the two groups: 545 patients were assigned to the calcium-channel blocker group and the rest to β blocker. 1.5-year survival rate was compared between groups. The result of the study is that there was no significant difference between the groups in the incidence of cardiovascular death, non-fatal MI, unstable angina, and non-fatal stroke. As 77% of the study participants received primary PCI, this finding suggests the little need of administering β blocker to patients undergoing successful primary PCI^17^. Furthermore, we should not ignore the side effect of β blocker in the present situation where the efficacy of β blocker is not clarified yet in STEMI patients undergoing primary PCI. The issue of adverse effects caused by β blocker is a concern. In the COMMIT trial, one of the largest trial enrolling approximately 46000 AMI patients, the intravenous then oral administration of metoprolol reduced the risk of re-infarction from 2.5 % to 2.0% and the risk of ventricular fibrillation from 3.0 % to 2.5 % in the metoprolol group as compared to the placebo group for up to 28 days in hospital, while it increased the risk of cardiogenic shock from 3.9 % to 5.0 % in the very acute period. As a result, there was no significant difference in 28-day all-cause mortality (average follow-up period; 14 days) between the groups (metoprolol group; 7.7%, placebo group 7.8%)^18^. This COMMIT trial was the first study which clarified the adverse effect of very early β blocker therapy and it was stressed that we needed to pay attention to the risk of cardiogenic shock if we chose intravenous administration of β blocker.

In Japan, it was reported that the prevalence of patients with vasospastic angina was high^17,19^. Hence, we should bear in mind the risk of anginal attack due to vasospasm worsened by the use of β blocker. Other commonly reported side effects of β blocker included hypotension, bradycardia, lightheadedness, bronchospasm, and peripheral circulatory failure, abnormal liver function, gastrointestinal symptoms, a feeling of weakness, depression, and impotent. Especially in aged people, falls due to orthostatic hypotension is one of the common clinical problems. Although the incidence of adverse effects by β blocker is not accurately known, it is necessary to confirm the significance of β blocker therapy for STEMI patients based on both risk and benefit. It is important to discuss whether β blocker is essential to STEMI patients from the viewpoint of the issue of the skyrocketing national medical care expenditure.

We, the cardiovascular research group of Kyoto University, asking for collaboration with other 30 hospitals, have built the system of the physician-initiated, multi-center clinical research and its data management. We have surveyed the treatment of Japanese coronary artery disease patents in daily practice, assessed the treatment outcomes and investigated factors influencing them^20,21^.

For example, J-Cypher Registry, which was a prospective multi-center registry enrolling consecutive patients undergoing sirolimus-eluting stent (CYPHER^®^) implantation without any exclusion, was conducted in collaboration with 37 participating centers. In the registry, 910 STEMI patients underwent primary PCI and only 394 of the patients (38.4%) received β blocker therapy at discharge. This data suggest a discrepancy between the treatment recommended by the guideline and that observed in real clinical practice concerning the role of β blocker in the early therapy for STMI patients in our country. In addition, 3-year survival rate of those 910 patients was 91.3% and there was no difference in the survival rate between the β blocker and the no-β blocker groups. However, there was a limit to the discussion of clinical efficacy of β blocker due to the observational nature of the J-Cypher Registry.

This multi-center, prospective, open-label randomized study aims to investigate the effect on 3-year mortality of β blocker therapy in STEMI patients with preserved LVEF and with little residual ischemia who underwent primary PCI. The result from this trial should be one of the landmarks, which guide our medical treatment for STEMI patients receiving revascularization as primary PCI.

**III．Trial plan**

1. **Study design**

Physician-initiated, multi-center, open label, randomized controlled study

1. **Study period and enrollment period**

Study period:

13/07/2010-12/07/2015 (For 5 years after the date of the ethical approval)

Enrollment period:

13/07/2010-12/07/2012（For 2 years after the date of the ethical approval）

1. **Selection of study participants**

3-1　Enrollment criteria and eligibility

3-1-1 Capturing the number of STEMI cases in each participating center

Each participating center reports the total number of STEMI patients and the total volume of primary PCI to the data management center.

3-1-2　Randomized controlled study

・If STEMI patients undergo successful revascularization by primary PCI within 24 hours after the onset and have preserve left ventricular ejection fraction (LVEF ≥40%) as assessed by echocardiograph, they will participate in this randomized controlled trial unless they meet the following exclusion criteria. They should be randomized within 7 days after primary PCI.

・Written informed consent has to be obtained from study participants.

※Definition of STEMI: chest symptoms at rest lasting longer than 20 minutes, a new or presumably new

change of ST-segment elevation of ≧1mm in ≧2 consecutive precordial leads or 2 contiguous leads in

limb leads and elevated myocardial biomarkers (higher than the upper limit of troponin T, troponin I or

CK-MB).

※※Definition of successful primary PCI: PCI within 24 hours from onset and recanalization of the target vessel.

3-2　Exclusion criteria

1. Contraindicated to β blocker therapy※
2. Reduced left ventricular function (LVEF＜40%)
3. Prior cardioverter defibrillator implantation
4. Considered not suitable for the study by the attending physician (Example: patients expected to live less than a year due to malignancy or other non-cardiac diseases, pregnant and breast-feeding patients)

※Patients contraindicated to β blocker therapy

1. Low output status
2. High risk of suffering from cardiogenic shock
3. Advanced first-degree atrioventricular block (PR interval＞240msec)
4. Second-degree and third-degree block
5. Have a sign of congestive heart failure
6. Have severe bronchial asthma or obstructive lung disease requiring medical therapy
7. Allergic to β blocker
8. **Enrollment**

4-1 Informed consent

The physician in charge (the attending physician) make a sufficient explanation including disadvantage about the trial, obtaining written informed consent from patients with STEMI who undergo primary PCI within 24 hours after the onset unless they meet any exclusion criteria. When the study candidates cannot communicate well, consent from their family or guardian is also acceptable. Informed consent and registration must be completed within 7 days after primary PCI.

4-2 Registration

After obtaining informed consent from the eligible patients in each participating center, we register them through a web-based database within 7 days after primary PCI.

4-3 Screening period

Screening period is defined as required time for randomization after obtaining informed consent. The study participants are randomized to the carvedilol group or to the no β blocker group within 7 days after the onset.

4-4 Randomization

Eligible patients are randomized to the carvedilol group or to the no β blocker group on the Web and initiate their assigned therapy. Randomization was pre-stratified by each participating center.

4-5 Concomitant pharmacological and non-pharmacological therapy

Not only concomitant pharmacological therapy but non-pharmacological therapy such as rehabilitation is left to the physician in charge in each participating center. A combination of Aspirin and clopidogrel (Plavix^®^) is recommended as dual antiplatelet agents in patients receiving stent implantation, but ticlopidine (Panaldine^®^) can be used. Management of blood pressure and cholesterol, glycemic control should be conducted according to each guideline. Information about concomitant medical therapy is put into the online baseline form. Patients who is already on β blocker, should discontinue the β blocker at the registration. The study participants can start or discontinue β blocker when the attending doctor judges it necessary for any clinical reason. In either cases, the crossover from one treatment strategy to the other should be reported to the study office as soon as possible.

1. **Intervention**

The only β blocker available in this study is carvedilol (Artist^®^/Artione^®^/Atenote^®^/Anist^®^). Carvedilol, a non-selective β blocker, also has an α1 receptor-blocking effect and is reported to be more effective than other β blocking agents for STEMI patients in overseas studies^11,22^. Carvedilol is the only β blocker which was shown to improve the prognosis of patients with cardiac disease in Japan, too^23^. Carvedilol has anti-oxidant, anti-inflammatory and cardioprotective action and is less harmful to carbohydrate and lipid metabolism than other β blockers^24^.

The drug might improve renal blood flow as a renal protective effect. It was demonstrated that even low-dose carvedilol was effective for Japanese people^23^. The initial and the maintenance dose of carvedilol is at the discretion of the attending physician. In principle, however, it should be initiated from low doses as always, and be up-titrated to the maximum dose of 20mg/day, which was approved by the national health insurance. Our trial does not adopt blinding because blinding with placebo in the studies with physician initiatives is practically very difficult in Japan.

1. **Follow-up**

6-1 Date of follow-up

Study participants are followed at 3 months, 12 months, 36 months after registration and at the completion of the study. Follow-up is basically by a hospital visit, but a telephone contact or exchange of letters is also approved if they cannot make a visit for some reasons. Collected data are entered into the online follow-up form by the physician in charge belonging to each participating center. The physician in charge should enter data within 7 days after each date of follow-up. The window period at 3 months is within 4 weeks of the date of follow-up and the window period at 12 and 36 months within 8 weeks of the date of follow-up. Refer to follow-up form about follow-up variables.

6-2 Completion of the study

The final number of event (the number of all-cause death) is confirmed when the 3-year follow-up of the final enrolled patient is completed. If the number of event in the no-β blocker group doesn’t reach 193, which was the calculated sample size required in this study, the data safety monitoring board will decide whether trial can be continued or not in accordance with interim analysis. Only if the board approves of the trial continuation, the study duration can be prolonged. The study is completed when 193 events in the no-β blocker are confirmed.

1. **Follow-up schedule**

7-1 Study schedule

**At the completion of the study**

**◆Carvedilol group**

**◆No-β blocker group**

**PCI**

**Concomitant medical therapy (left to the attending doctor)**

**Concomitant medical therapy (left to the attending doctor)**

No β blocker therapy in principle

**Low dose of carvedilol～the maximum dose of 20 mg/day**

**Randomization**

**3M**

**PCI**

**12M**

**36M**

7-2 Schedule of follow-up assessment and data collection

※CCS grading system; the degree of anginal symptom defined by Canadian Cardiovascular Society. The severity of exertional angina is categorized into the following 4 types.

CCS class I

Ordinary physical activity causes no angina, such as walking and climbing stairs. Angina with strenuous or rapid or prolonged exertion at work or recreation.

CCS class II

Slight limitation of ordinary activity. Walking or climbing stairs rapidly, walking uphill, walking or stair climbing after meals, or in cold or in wind, or under emotional stress, or only during the few hours after awakening. Walking more than two blocks on the level and climbing more than one flight of ordinary stairs at a normal pace and in normal conditions.

CCS class III

Marked limitation of ordinary physical activity. Walking one or two blocks on the level and climbing one flight of stairs in normal conditions and at normal pace.

CCS IV

Inability to carry on any physical activity without discomfort, anginal syndrome may be present at rest.

※※Killip classification: grading system assessing the severity of left-sided heart failure associated with acute myocardial infarction based on physical findings. The severity of heart failure was ranked into the following 4 types.

Killip classⅠ　No sign of pump failure No heart failure

Killip classⅡ　Rales in 1/2 of the lung field　Mild heart failure(third heart sound, elevated jugular venous pressure)

Killip classⅢ　Rales in the lung field　　 Pulmonary edema

Killip classⅣ　Cardiogenic shock　　　　 Cardiogenic shock

※※※NYHA classification：the grading system for classifying the severity of symptoms due to heart failure defined by New York Heart Association (NYHA). The severity of heart failure is subclassified into the following 4 types.

NYHA class I

No limitations of physical activity. Asymptomatic left ventricular dysfunction.

NYHA class II

Mild to moderate limitations of physical activity. No symptom at rest, but ordinary physical activity results in fatigue, palpitation, dyspnea or angina.

NYHA class III

Marked limitation of ordinary physical activity. Less-than-ordinary physical activity such as walking short distances causes symptoms.

NYHA class IV

Unable to carry on any physical activity without symptoms of heart failure or angina pectoris. Symptoms at rest might occur.

※Check-up of compliance

1) The attending physician at each participating center asks the study participant of the medication compliance and writes it down according to the following criteria. The physician also puts the information into a questionnaire.

Criteria about the compliance of carvedilol

| 1 | Take medication as suggested (More than 95%) |
| --- | --- |
| 2 | Sometimes forget to take medication (More than 80% to less than 95%) |
| 3 | Often forget to take medication (More than 40% to less than 80%) |
| 4 | Always forget to take medication (less than 40%) |

2) The presence of concomitant medication, each drug name, daily dosage, the period of concomitant use

3) Investigation about medical self-management by direct interview or with a questionnaire.

1. **Dropout or termination of the study**

　The discontinuation of carvedilol because of side effects caused by carvedilol or for a certain reason in the carvedilol group should be reported to the data center by the physician in charge as soon as possible. Similarly, the unexpected initiation of β blocker of patients in the no-β blocker should also be reported to the data center. In these crossover cases, follow-up survey about clinical events should also be continued until the completion of the study. Furthermore, patients who withdrew consent are regarded as dropout and the intention-to-treatment analysis is conducted after exclusion of such dropouts.

1. **Statistical analysis**

9-1 Definition of endpoint

The following endpoints are adjudicated by the independent clinical event committee

9-1-1 Primary endpoint

1) Death from any cause (all-cause death)

2) A composite of all-cause death, myocardial infarction, hospitalization for acute coronary syndrome, and hospitalization for congestive heart failure

9-1-2 Secondary endpoint：evaluated in the hierarchical order

1) Cardiac death

2) Sudden cardiac death

3) Cardiovascular death

4) Myocardial infarction (MI)

5) Hospitalization for acute coronary syndrome

6) Persistent ventricular tachycardia and ventricular fibrillation

7) Hospitalization for congestive heart failure

8) ARC-definite stent thrombosis

9) Any target-lesion revascularization

10) Any clinically-driven target lesion revascularization

11) Any coronary revascularization

12) Any clinically-driven coronary revascularization

13) CABG

14) Stroke

15) Vasospastic angina

16) Major bleeding

17) A composite of all-cause death, MI, stroke, hospitalization for acute coronary syndrome, hospitalization for congestive heart failure, and any coronary revascularization

18) A composite of cardiac death, MI, hospitalization for acute coronary syndrome, and hospitalization for congestive heart failure

19) A composite of cardiovascular death, MI, and stroke

9-1-3 Definition of clinical event

9-1-3-1 Death

・Cardiac death

Includes in-hospital death after registration, sudden death, death for unknown, unidentified or unexplainable cause, death due to heart failure, myocardial infarction and PCI or CABG, and death related to cardiac procedures associated with infection and so on.

・Sudden cardiac death

Death within 24 hours of the symptom onset in stable patients, death during the sleep or unwitnessed death.

Sudden cardiac death can be denied if obvious non-cardiac cause is identified. Extrinsic cardiac death such as traffic accident does not belong to this category.

・Non-cardiac death

Death unrelated to heart disease such as malignancy, pneumonia, suicide, accident, cerebrovascular disease, aortic aneurysm

・Cardiovascular death

Includes not only cardiac death, but death due to cerebrocardiovascular death such as cerebral infarction and hemorrhage, peripheral artery disease, chronic kidney disease, aortic aneurysm/dissection.

・Arrhythmic death

Includes sudden cardiac death and death manly due to arrhythmia

The concrete example is death that occurs 24 hours after the resuscitation attempted for life-threatening arrhythmia not related to AMI.

・Heart failure death

Death mainly due to heart failure

・Acute coronary syndrome (ACS) death

Death likely to be caused by ACS, where several findings such as ST-elevation in ECG or elevated enzymes indicate the diagnosis of ACS

・Coronary artery disease death

Cardiac death except for death clearly unrelated to coronary artery disease such as heart failure death due to valvular disease or dilated cardiomyopathy.

9-1-3-2 Myocardial Infarction (MI)

MI is classified according to the ARC definition. Procedure-related MI, however, is evaluated by the value of CK-MB but not by troponin because of very high sensitivity.

1. Diagnosis of preprocedural MI

ST-elevation in ECG, new abnormal Q wave, clinical symptoms typical of MI, higher than the upper limit of troponin T, troponin I or CK-MB

1. Periprocedural MI
   - Any one of the following findings detected within 48 hours after PCI meets the diagnosis of MI.

- Three or more times the upper limit of normal value of CK-MB（defined as MI at registration but not new-onset MI when the preprocedural myocardial enzyme is higher than the upper limit of normal.）
  - - - Abnormal electrocardiographic findings [new abnormal Q wave, left bundle branch block (LBBB)]
  - When the value of CK-MB, Troponin T or Troponin I measured within 72 hours after CABG is five or more times the upper limit of normal value, any one of the following criteria meets the diagnosis of MI (defined as MI at registration, but not new-onset MI when the preprocedural myocardial enzyme is higher than the upper limit of normal value).
    - - Abnormal electrocardiographic findings (new or presumably new-onset abnormal Q wave, LBBB)
      - New coronary artery or graft occlusion
      - Imaging evidence of new loss of viable myocardium.

1. Spontaneous MI
   - - Any one of the following criteria meets the diagnosis of MI 48 hours after PCI or 72 hours after CABG. The definition of procedure-related MI is applied to PCI-related MI such as TLR and TVR.
       - Abnormal electrocardiographic findings (new or presumably new-onset abnormal Q wave, LBBB)

- Higher than the upper limit of troponin T, troponin I, or CK-MB (defined as MI at registration but not new-onset MI when the preprocedural myocardial enzyme is higher than the upper limit of normal value).

1. Sudden Death
   - - The diagnosis of MI follows the following criteria in cases, who died presumably on a rise of cardiac biomarker value or before measurement of the biomarker value.
       - Detection of any one of the following findings in addition to symptoms suggestive of ischemia
         - New or presumably new-onset ST-elevation or LBBB
         - Identification of an intracoronary thrombus by angiography or autopsy
2. Reinfarction
   - - Diagnosed under the following condition: two times measuring cardiac biomarker value after the onset of MI detects a rise and/or fall of the value but the value of cardiac biomarker measured in 3-6 hours is elevated by 20 percent of the value.
       - The diagnosis of reinfarction is impossible on a rise of cardiac biomarker values or before the peak of the value.

ECG criteria：

1. Q wave
   - - QMI
       - Abnormal Q waves in 2 contiguous leads irrespective of elevation of myocardial enzyme
     - Non-Q MI
       - MI other than QMI
2. ST-T segment
   - - ST-elevation MI
       - New or presumably new ST elevation at the J point in two contiguous leads with the cutoff-points: ≥ 0.2 mV in V1-V3 and ≥ 0.1 mV in the other leads.
     - Non ST-elevation MI
       - MI other than STEMI

9-1-3-3 Persistent ventricular tachycardia/ventricular fibrillation

Ventricular tachycardia and ventricular fibrillation requiring defibrillation or intravenous administration of anti-arrhythmic agents

9-1-3-4 Coronary revascularization

1. Target Lesion Revascularization (TLR)

PCI for target lesion（a lesion within the stent or the 5-mm borders proximal or distal to the stent）or CABG for restenosis of target lesion or other complications

- - Target Vessel Revascularization (TVR)

Revascularization of target vessel by PCI or CABG. TVR includes TLR.

- - - 1. Clinically indicated revascularization

1. Any one of the following criteria meets clinically indicated revascularization. The operator confirms the presence or absence of clinical findings.
   - - Angina recurrence presumably due to target vessel
     - Ischemia due to target vessel at rest or confirmed by stress test
     - Functional ischemia detected invasive modality [Doppler flow velocity reserve (FVR), Fractional flow reserve (FFR)]

9-1-3-6 Stent Thrombosis

On the basis of the ARC definition, stent thrombosis (ST) is subcategorized into definite, probable, and possible ST by probability or into acute, subacute, late and very late ST by the timing of onset.

・Definite ST

The following criteria meets the diagnosis: the setting of acute coronary syndrome and detection of acute intracoronary thrombosis or occlude coronary artery by angiography or autopsy.

・Probable ST

Death for unknown cause within 30 days of stent implantation (including sudden death) or myocardial infarction probably due to the occlusion of target vessel without confirmation by angiography or detection of culprit vessel

・Possible ST

Death for unknown cause beyond 30 days after stent implantation (including sudden death)

Any one of the following criteria meets the diagnosis of stent thrombosis.

＊Angiographic occlusion of coronary artery without clinical ischemic findings doesn’t satisfy the diagnosis of stent thrombosis (silent occlusion).

・Acute Stent Thrombosis

0～24 hours after the procedure（0 hour is defined as the time of removing the guiding catheter）

・Subacute Stent Thrombosis

24 hours～30 days after the procedure

・Late Stent Thrombosis *

31 days～1year after the procedure

・Very Late Stent Thrombosis *

Beyond 1 year

9-1-3-7 Emergency hospitalization for ACS

Myocardial infarction or unstable angina pectoris (UAP) requiring emergency hospitalization whether it is categorized into Braunwald Ⅲ type or not.

(The following situation is not included here: suspicion of UAP results in emergency hospitalization but culprit vessel is not identified in the coronary angiography.)

9-1-3-8 Hospitalization for congestive heart failure

Hospitalization based on the diagnosis of worsened heart failure requiring intravenous treatment. This includes a hospitalized case requiring intravenous treatment for worsening heart failure

9-1-3-9 Bleeding/Hemorrhagic Complications

Evaluated according to the definition of GUSTO^14^。

GUSTO Bleeding Classification:

Severe Bleeding

- Life-threatening hemorrhage

1. Intracerebral hemorrhage
2. Results in hemodynamic compromise requiring massive fluid, blood transfusion, catecholamine and surgical

procedure

Moderate Bleeding

1. Bleeding which requires blood transfusion but does not meet criteria for severe bleeding

※Bleeding events do not comprise autologous transfusion.

9-1-3-10 Stroke or Cerebrovascular Accident

All of the following findings meet the diagnosis: acute-onset neurological disorder, symptoms lasting longer than 24 hours, and the disturbance of cerebrovascular circulation by hemorrhage or ischemia. Transient ischemic attack, where the symptom vanished within 24 hours after the onset, is not included.

9-1-3-10 Worsening of vasospastic angina

The diagnosis is defined by worsening of angina at rest and treatment with more nitrate or calcium-channel blocker. The following case is not included here: A new stenotic lesion is confirmed by angiography and revascularized.

- 1. Calculation of sample size

In this study, STEMI patients undergoing successful primary PCI were randomized to the carvedilol group or to the no-β blocker group, where a composite of all-cause mortality, MI, hospitalization for ACS, and hospitalization for HF (MACE) was set as the primary endpoint. From the result of 14.0 % MACE at 3-year in STEMI patients undergoing primary PCI in the j-cypher registry, 30,1% MACE at 6-year in STEMI patients who underwent successful primary PCI and survived to discharge was expected. Then, based on the hypothesis of a relative 23% reduction of MACE in the carvedilol group^11^, the target sample size was calculated based on the following settings:

・ Two tailed test

・ Two-tailed alpha = 0.05

・ Power = 80% (β= 0.2)

From the calculation, at least 639 subjects in each arm were considered necessary. Then a total of 1300 patients (650 patients in each arm) were to be enrolled considering possible dropout (2 percent of the total study population) during follow-up.

9-3 Main Analysis

9-3-1 Descriptive statistics

Descriptive statistics is conducted about the following variables.

1. Ratio of the number of STEMI patients undergoing primary PCI to all the STEMI patients, and ratio of the number of the study participants to all the STEMI patients

(2) Patient background in all the study participants and patient background in STEMI patients belonging to each group

(3) Frequency and reason of discontinuation of carvedilol in the carvedilol group

(4) Frequency and reason of initiation of β blocker in the no-β blocker group

9-3-2 Superiority test of β blocker

Univariate survival analysis is performed in the primary and the secondary endpoint.

9-3-3 Independent predictors of adverse event

Analysis will be conducted as to the frequency of adverse event observed in the carvedilol group and the independent predictors

9-4 Prespecified subgroup analysis

The following subgroup analysis is conducted:

9-4-1 LVEF≧50% and <50%

9-4-2 Age (median)

9-4-3 Diabetes mellitus

9-4-4 Statin therapy

9-4-5 EPA/AA ratio≦0.55, EPA/AA ratio 0.55-1.06, EPA/AA≧1.06 (all evaluated in the acute phase )

9-4-6 Association between the final dose of carvedilol (≧ and < median of the dose ) and its prognosis

9-4-7 Participation in cardiovascular rehabilitation program in the acute and recovery period

9-5 Substudy

9-5-1 Substudy A：Association between frequency of CYP2c19 polymorphism and the prognosis

9-5-2 Substudy B：Substudy using echocardiography

9-5-2 Substudy C：Substudy using cardiac MRI

9-6 Analysis method

Analysis is made by intention-to-treat principle in the full analysis set of all the randomized participants irrespective of medication they really take, their compliance and change of the regimen. Moreover, separate analysis is made in the crossover cases. The α level is set as two-tailed 0.05 and confidence interval (CI) set as two-tailed 95% CI.

9-7 Interim analysis

For assessment of the safety, the independent safety committee will perform an interim analysis at 1-year follow-up of the last enrolled patient. The decision on continuation of the trial is based on α spending function.

9-8 Handling of missing data

In principle, missing values are regarded as missing and they are not complemented. How to handle the data of endpoints is discussed and decided in the clinical conference.

1. **Adverse event**

10-1 Definition of adverse event and its handling

An adverse event is any unfavorable or unexpected sign (including abnormal laboratory finding), symptom, and disease occurring in a patient administered an investigational product whether it has a causal relationship with the product or not. When serious adverse events are developed in the study duration, concomitant suspect medications should be reported immediately to the director in the participating center and the clinical research center regardless of type of adverse event, date of event, its severity, action taken regarding the study drug, outcome, date of outcome, a causal relationship with β blocker. The physician in charge should register on the website content of event, date of occurrence and disappearance, dose at the timing of occurrence of event, type, extent, action taken regarding the study drug, treatment for the event, outcome, a causal relationship with the product, reason for the decision about the causal relationship. It is regarded as side effect if the negative relationship with the study drug cannot be confirmed.

10-2 Unanticipated adverse event

As it is reported that the prevalence of vasospastic angina is high in Japanese patients, administration of β blocker might cause anginal attack. However, in JBCMI study, reported by Ogawa et al. in Kumamoto University, 1090 patients who had AMI within 30 days after the onset were randomized in a 1 to 1 fashion to the calcium-channel blocker group and the β blocker group and they are compared in mortality at 1.5-year follow up. In conclusion, there was no statistical difference between the groups in the incidence of cardiovascular death, non-fatal MI, UAP and non-fatal stroke^17^. It is reported that common side effects by β blocker other than vasospastic angina include hypotension, bradycardia, dizziness, bronchospasm, peripheral circulation failure, severer hypoglycemia attack, depression, impotence. These possible side effects should be meticulously surveyed and the investigation and the report are conducted about frequency, severity, management, outcome, causal relationship.

10-3 Action taken against serious adverse effect

A serious adverse event is defined as any untoward occurrence at the timing of use of the medical product or device, which meets that following criteria;

1. Resulting in death
2. Life-threatening
3. Requires hospitalization for treatment or prolonged hospitalization
4. Results in persistent or significant disability or incapacity
5. Leads to congenital anomaly

In this study performed in Kyoto University Hospital, the report and the management of serious adverse events conforms to “Report and Management Manual For Serious Adverse Event” and “Report and Management Flowchart For Serious Adverse Event in Clinical Study (investigation)”, which was specified separately by Kyoto University Hospital.

When adverse events occur in relation to this clinical study and the study subject suffer from health damage, the physician in charge or the attending doctor in each participating center should take the best measure for it such as quick and adequate medication. However, the health insurance will apply to the medical bill and medical compensation will not be given.

**11. Payment and insurance**

11-1 Compensation for the subject in the study-related injury and insurance

As β blocker therapy is all within the range of daily clinical practice and covered by health insurance, adverse reaction associated with the treatment will be compensated for according to the adverse drug reaction relief system. Compensation for other injury caused by this study is only provided if legal negligence liability arises. As it is assumed that the risk of the study-related injury is extremely low, the study office does not buy liability insurance for compensation. The injury not associated with this study but resulting from medial practice itself will be left at the discretion at each participating site.

11-2 Payment for medical expenses and transportation cost

The β blocker therapy in this study is covered by health insurance. No payment for medical expenses, transportation fee, and so on.

1. **Ethical issues**

The study doctors will conduct this study in accordance with ethical principles based on Declaration of Helsinki: they follow the ethical guideline about the clinical study of the Ministry of Health and Welfare and conform to the principles of GCP to protects the right and welfare of subjects and secure the scientific quality, the reliability, and the safety of the study. The attending physician and the study collaborators pay much attention to confidentiality about subjects (the identification of subjects by their ID number, the management of examination record and informed consent form).

12-1 **Explanation to the subject**

Prior to the enrollment, the study doctor shall hand over the explanation document approve in the ethics committee to the subjects and orally inform them of the details described in the documents. After the explanation, write down the required information in the Informed Consent Form attached to the explanation document and have the subjects sign. After filling in the required information in the Informed Consent From, two copies should be produced, one for the subject and one for the doctor, and the original version should be stored with Cardiovascular Clinical Research Promotion Department of Kyoto University.

12-2 **Items related to Privacy**

The medical record, laboratory data, and records related to the informed consent of the subjects and the like, and case report form and the related documents should be collected and stored in the Registration and Data Center at Department of Cardiovascular Internal Medicine, Kyoto University Hospital.

The storage site is on the shelf whose door can be locked, and the key shall be stored by the deputy principal investigator, who is responsible for the management of subjects’ privacy. The electronic files created for analysis shall be stored in the form of the memory media such as CD. They should be similarly stored in the shelf, which becomes locked. They should not be stored in the hard disk of the computer except when they are used for analysis. These records may be disclosed as required by audit request but they will be kept confidential. Also, these records must be stored in the way to allow easy searching. All personnel involved in this study have the confidentiality obligation as the data handling personnel, and therefore they in general must make their every effort to protect personal information.

**13. Approval and revision of protocol**

13-1 Approval of protocol

This study shall be conducted after the protocol is assessed and approved by the ethical committee in Kyoto University Hospital.

13-2 Revision of protocol

When revision of the protocol is required after the implementation of the protocol, the survey should temporarily be discontinued and the revised protocol is assessed and approved by the ethical committee again.

**14. Discontinuation and Termination of the study**

The study in principle shall be continued until the target number of subjects is registered and the evaluationforall the subjects is completed. However, when any adverse events that are cleraly related to this study occur, the independent safety evaluation committee shall discuss whether the study should be continued.

14-1 Interim report to the ethics committee

As the study period is supposed to be 8 years, the study progress report should be submitted to the ethical committee 4 years after the initiation whether the study is continued or not.

14-2 Discontinuation of the study

When any reasons compelling the study discontinuation arise, the principal investigator shall, after discussion with the facilitator’s committee, immediately report the discontinuation of the study and the reason for the discontinuation to each site’s ethical committee in a written document.

14-3 Termination of the study

The principal investigator shall inform the primary investigator of each site of the completion of the subject enrollment when the enrollment of all the subjects is completed. Then each site shall discontinue the subject enrollment. The principal investigator shall also inform the completion of subject follow-up of the primary investigator of each site when the completion of follow-up of all the subjects is confirmed. The primary investigator of each site shall submit the completion report to the director of each medial research.

**15. Presentation of the result and publication**

The presentation and the publication of this study will be performed after discussion among the principal investigator, the steering committee and the person in charge for statsitical analysis. Evaluation of the result of this study will be seeked in the national and international scientific meeting about cardiovascular medicine and be disclosed to the society through each media. Whenever the participating centers request the analysis of their own data, it will be offered in the form of excel sheet. The result of the interim analysis will not be discosed except when the safety is questioned.

In publication of the study result, the study funder is disclosed. Regrettably, the name of some study doctors cannot be listed as co-author in this publication. After confirming their cooperation in the study, their name and affilicated center are individually described as acknowledgement within the limit by the journal and the society.

**16. Handling of the study result after publication**

The principal investigator and the steering committee are not resticted on the handling of the study result after publication. The main study funder can use the copyrighted material such as the slides used in the presentation of the study result and the accepted paper because of the same right as the authors have (the use approved by the auhors should be stipulated when necessary).

**17. Funding source**

Grant-in-Aid for Scientific Research of The Ministry of Education, Culture, Sports, Science, and Technology applied for. No conflict of interest.

**18. Study Organization**

Principal study site

Department of Cardiovascular Medicine, Graduate School of Medicine and Faculty of Medicine Kyoto University

Principal investigator

Department of Cardiovascular Medicine, Graduate School of Medicine and Faculty of Medicine Kyoto University

Professor Takeshi Kimura

Shogoin Kawahara-cho 54, Sakyo-ward, Kyoto-city, 606-8507

Phone: 075-751-4254 FAX: 075-751-3289

Depty principal investigator

Department of Cardiovascular Medicine, Graduate School of Medicine, Faculty of Medicine Kyoto University

　　Assistant Professor　　　　　Neiko Ozasa

Shogoin Kawahara-cho 54, Sakyo-ward, Kyoto-city, 606-8507

Phone: 075-751-4255 FAX: 075-751-3299

**Steering Committee:**

Shizuoka General Hospital　　　　 Hiroshi Nonogi

Kokura Memorial Hospital　　　　　　　　　　　　　　　　Masakiyo Nobuyoshi

Kurashiki Central Hospital　　　　　　　　　　 Kazuaki MItsudo

Tsuchiya General Hospital　　　　　　　　　　　 Yasuhiko Hayashi

Saiseikai Kumamoto Hospital　　　　　　　　 Koichi Nakao

Sakakibara Memorial Hospital　　　　　　　　　 Tetsuya Sumiyoshi

Toyohashi Heart Center　　　　　　　　　　　　　 Takahiko Suzuki

Hokkaido Social Insurance Hospital　　　　　　　　　　 Keiichi Igarashi

Teikyo University Hospital　　　　　　　　　　　　　　　 Isshiki Takaaki

Saint hirukai　　　　　　　　　　　 Masunori Matsuzaki

Yokohama City University Medical Center　　　　　　　　 Kazuo Kimura

Kindai University Hospital　　　　　　　　　　　　　　 Shunichi Miyazaki

Shiga University Of Medical Science Hospital　　　　 Minoru Horie

Kobe University Hospital　　　　　　　　　　　　　　　 Kenichi Hirata

Okayama University Hospital　　　　　　　　　　　　　　　Hiroshi Ito

Nagoya University Hospital　　　　　　　　　　　　　 Toyoaki Murohara

Tokai University Hospital　　　　　 Yuji Ikari

National Cerebral and Cardiovascular Center Masaharu Ishihara

Study Sites（Plan）

Kyoto University Hospital

Plan to request other 320 centers for participation.

**Clinical Event Committee**

This study will set the case conference committee as the committee organization independent of the study organization. The clinica event committee shall evaluate the content of srious adverse events which occurred in the study period and categorize each endpoint.

Tenri Hospital　　　　　　　　　　　　　　　　 Yoshihisa Nakagawa

National Hospital Organization Kyoto Medical Center　 Masaharu Akao

Kobe City Medical Center General Hospital　　　　　 Yutaka Furukawa

**Data and Safety Monitoring Board**

This study will set the Data and Safety Monitoring Board (DSBM) as the committee organization independent of the study organization. The DSMB should assess, review and analyze accumulated the study data such as mainly for subject safety. Then the DSMB will make recommendations to the steering committee concerning the continuation and termination of the study from the scientific and the ethical viewpoint.

Kumamoto University Hospital　　　　　　　　　　 Hisao Ogawa

Toranomon Hospital　　　　　　　　　　　　　　 Tetsu Yamaguchi

**Study Secretariat**

Research Institute for Production Development

Shimogamo-morimoto-cho 15, Sakyo-ward, Kyoto-city, 606-0805

Phone: 075-781-1107　　　　Fax: 075-791-7659

Person in charge of the study: Cardiovascular Clinical Research Promotion Department

Person in charge: Kumiko Kitagawa

**Data Center**

Department of the Cardiovascular Medicine, Graduate School of Medicine and Faculty of Medicine, Kyoto University

Shogoin Kawahara-cho 54, Sakyo-ward, Kyoto-city, 606-8507

Phone: 075-751-4068 Fax: 075-751-3037

Person in charge of database

Department of the Cardiovascular Medicine, Kyoto University　　 Neiko Ozasa

Person in charge for statistical analysis

The responsible statistician will develop an idea aboout analysis paln, make an analysis plan document, and make a statistical analysis or discussion about the analysis of efficacy and safety data.

Faculty of Medicine, Kinki University　　　　　 Takeshi Morimoto

Substudy A Core Lab Person in charge

Department of the Cardiovascular Medicine, Kyoto University　 Takeshi Makiyama

Substudy B Core Lab Person in charge

Department of Cardiology, Tenri Hospital　　　　　　　　　 Chisato Izumi

Substudy C Core Lab Person in charge

　　Kobe City Medical Center General Hospital　　　 Shuichiro Kaji

1. **Reference**

1. De Luca G, Suryapranata H, Ottervanger JP, Antman EM. Time Delay to Treatment and Mortality in Primary Angioplasty for Acute Myocardial Infarction: Every Minute of Delay Counts. Circulation 2004;109(10):1223–5.

2. ISIS Study Group. Randomised trial of intravenous atenolol among 16 027 cases of suspected acute myocardial infarction: ISIS-1. First International Study of Infarct Survival Collaborative Group. Lancet 1986;2(8498):57–66.

3. β-Blocker Heart Attack Study Group. The beta-blocker heart attack trial. beta-Blocker Heart Attack Study Group. JAMA 1981;246(18):2073–4.

4. Norwegian Multicenter Study Group. Timolol-induced reduction in mortality and reinfarction in patients surviving acute myocardial infarction. N Engl J Med 1981;304(14):801–7.

5. beta-blocker Heart Attack Trial Research Group. A randomized trial of propranolol in patients with acute myocardial infarction. I. Mortality results. JAMA 1982;247(12):1707–14.

6. Roberts R, Rogers WJ, Mueller HS, et al. Immediate Versus Deferred Beta-Blockade Following Thrombolytic Therapy in Patients With Acute Myocardial Infarction: Results of the Thrombolysis in Myocardial Infarction (TIMI) II-B Study. Circulation 1991;83(2):422–37.

7. Van De Werf F, Janssens L, Brzostek T, et al. Short-term effects of early intravenous treatment with a beta-adrenergic blocking agent or a specific bradycardiac agent in patients with acute mycardial infarction receiving thrombolytic therapy. J Am Coll Cardiol 1993;22(2):407–16.

8. Pfisterer M, Cox JL, Granger CB, et al. Atenolol use and clinical outcomes after thrombolysis for acute myocardial infarction: The GUSTO-I experience. J Am Coll Cardiol 1998;32(3):634–40.

9. Freemantle N, Cleland J, Young P, Mason J, Harrison J. Beta Blockade after myocardial infarction: Systematic review and meta regression analysis. Br Med J 1999;318(7200):1730–7.

10. Group T-IS. Comparison of invasive and conservative strategies after treatment with intravenous tissue plasminogen activator in acute myocardial infarction. Results of the thrombolysis in myocardial infarction (TIMI) phase II trial. N Engl J Med 1989;320:618–27.

11. Dargie HJ. Effect of carvedilol on outcome after myocardial infarction in patients with left-ventricular dysfunction: the CAPRICORN randomised trial. Lancet 2001;357(9266):1385–90.

12. Packer M, Coats AJ, Fowler MB, et al. Effect of carvedilol on survival in severe chronic heart failure. N Engl J Med 2001;344(22):1651–8.

13. Packer M, Bristow MR, Cohn JN, et al. The Effect of Carvedilol on Morbidity and Mortality in Patients with Chronic Heart Failure. N Engl J Med 1996;334(21):1349–55.

14. Antman EM, Anbe DT, Armstrong PW, et al. ACC/AHA guidelines for the management of patients with ST-elevation myocardial infarction: a report of the American College of Cardiology/American Heart Association Task Force on Practice Guidelines (Committee to Revise the 1999 Guidelines for the Managem. Circulation 2004;110(9):e82-292.

15. Guidelines for the management of patients with ST-elevation myocardial infarction (JCS2008). Circ J 2008;72(suppl.Ⅳ):1347–442.

16. Kasanuki H, Honda T, Haze K, et al. A large-scale prospective cohort study on the current status of therapeutic modalities for acute myocardial infarction in Japan: Rationale and initial results of the HIJAMI Registry. Am Heart J 2005;150(3):411–8.

17. Japanese beta-Blockers and Calcium Antagonists Myocardial Infarction (JBCMI) Investigators. Comparison of the effects of beta blockers and calcium antagonists on cardiovascular events after acute myocardial infarction in Japanese subjects. Am J Cardiol 2004;93(8):969–73.

18. Chen Z, Xie J. Early intravenous then oral metoprolol in 45 852 patients with acute myocardial infarction: Randomised placebo-controlled trial. Lancet 2005;366(9497):1622–32.

19. Pristipino C, Beltrame JF, Finocchiaro ML, et al. Major racial differences in coronary constrictor response between japanese and caucasians with recent myocardial infarction. Circulation 2000;101(10):1102–8.

20. Kimura T, Morimoto T, Furukawa Y, et al. Long-term outcomes of coronary-artery bypass graft surgery versus percutaneous coronary intervention for multivessel coronary artery disease in the bare-metal stent era. Circulation 2008;118(14 Suppl).

21. Kimura T, Morimoto T, Nakagawa Y, et al. Antiplatelet therapy and stent thrombosis after sirolimus-eluting stent implantation. Circulation 2009;119(7):987–95.

22. Kopecky SL. Effect of Beta Blockers, Particularly Carvedilol, on Reducing the Risk of Events After Acute Myocardial Infarction. Am J Cardiol 2006;98(8):1115–9.

23. Hori M, Sasayama S, Kitabatake A, et al. Low-dose carvedilol improves left ventricular function and reduces cardiovascular hospitalization in Japanese patients with chronic heart failure: The Multicenter Carvedilol Heart Failure Dose Assessment (MUCHA) trial. Am Heart J 2004;147(2):324–30.

24. Bakris GL, Fonseca V, Katholi RE, et al. Metabolic Effects of Carvedilol vs Metoprolol in Patients With Type 2 Diabetes Mellitus and Hypertension. JAMA 2004;292(18):2227.
